# Keys to Lipid Selection in Fatty Acid Amide Hydrolase Catalysis: Structural Flexibility, Gating Residues and Multiple Binding Pockets

**DOI:** 10.1371/journal.pcbi.1004231

**Published:** 2015-06-25

**Authors:** Giulia Palermo, Inga Bauer, Pablo Campomanes, Andrea Cavalli, Andrea Armirotti, Stefania Girotto, Ursula Rothlisberger, Marco De Vivo

**Affiliations:** 1 Laboratory of Molecular Modeling and Drug Discovery, Istituto Italiano di Tecnologia, Genova, Italy; 2 CompuNet, Istituto Italiano di Tecnologia, Genova, Italy; 3 Laboratory of Computational Chemistry and Biochemistry, Institute of Chemical Sciences and Engineering, École Polytechnique Fédérale de Lausanne, Lausanne, Switzerland; 4 Department of Pharmacy and Biotechnology, University of Bologna, Bologna, Italy; 5 D3-PharmaChemistry, Istituto Italiano di Tecnologia, Genova, Italy; UNC Charlotte, UNITED STATES

## Abstract

The fatty acid amide hydrolase (FAAH) regulates the endocannabinoid system cleaving primarily the lipid messenger anandamide. FAAH has been well characterized over the years and, importantly, it represents a promising drug target to treat several diseases, including inflammatory-related diseases and cancer. But its enzymatic mechanism for lipid selection to specifically hydrolyze anandamide, rather than similar bioactive lipids, remains elusive. Here, we clarify this mechanism in FAAH, examining the role of the *dynamic paddle*, which is formed by the gating residues Phe432 and Trp531 at the boundary between two cavities that form the FAAH catalytic site (the “membrane-access” and the “acyl chain-binding” pockets). We integrate microsecond-long MD simulations of wild type and double mutant model systems (Phe432Ala and Trp531Ala) of FAAH, embedded in a realistic membrane/water environment, with mutagenesis and kinetic experiments. We comparatively analyze three fatty acid substrates with different hydrolysis rates (anandamide > oleamide > palmitoylethanolamide). Our findings identify FAAH’s mechanism to selectively accommodate anandamide into a multi-pocket binding site, and to properly orient the substrate in pre-reactive conformations for efficient hydrolysis that is interceded by the *dynamic paddle*. Our findings therefore endorse a structural framework for a lipid selection mechanism mediated by structural flexibility and gating residues between multiple binding cavities, as found in FAAH. Based on the available structural data, this exquisite catalytic strategy for substrate specificity seems to be shared by other lipid-degrading enzymes with similar enzymatic architecture. The mechanistic insights for lipid selection might assist de-novo enzyme design or drug discovery efforts.

## Author Summary

We describe a new structural enzymatic framework to regulate substrate specificity in lipid-degrading enzymes such as fatty acid amide hydrolase (FAAH), a key enzyme for the endocannabinoid lipid signaling that hydrolyzes a variety of lipids, however with different catalytic rates. The identified novel mechanism and key features for lipid selection in FAAH are then analysed in the context of other relevant lipid-degrading enzymes. Through the integration of microsecond-long molecular dynamics simulations with mutagenesis and kinetic experiments, our study suggests that structural flexibility, gating residues and multiple cavities in one catalytic site are keys to lipid selection in the endocannabinoid system. Our results suggest that the structural framework proposed here could likely be a general enzymatic strategy of other lipid-degrading enzymes to select the preferred lipid substrate within a broad spectrum of biologically active lipids. This new, and likely general, structural framework for lipid selection in FAAH could therefore now encourage additional experimental verifications of the role of ligand and structural flexibility, as regulated by key gating residues at the boundaries of multiple cavities forming a single catalytic site, as observed in several other lipid-degrading enzymes.

## Introduction

**Fig 1:**
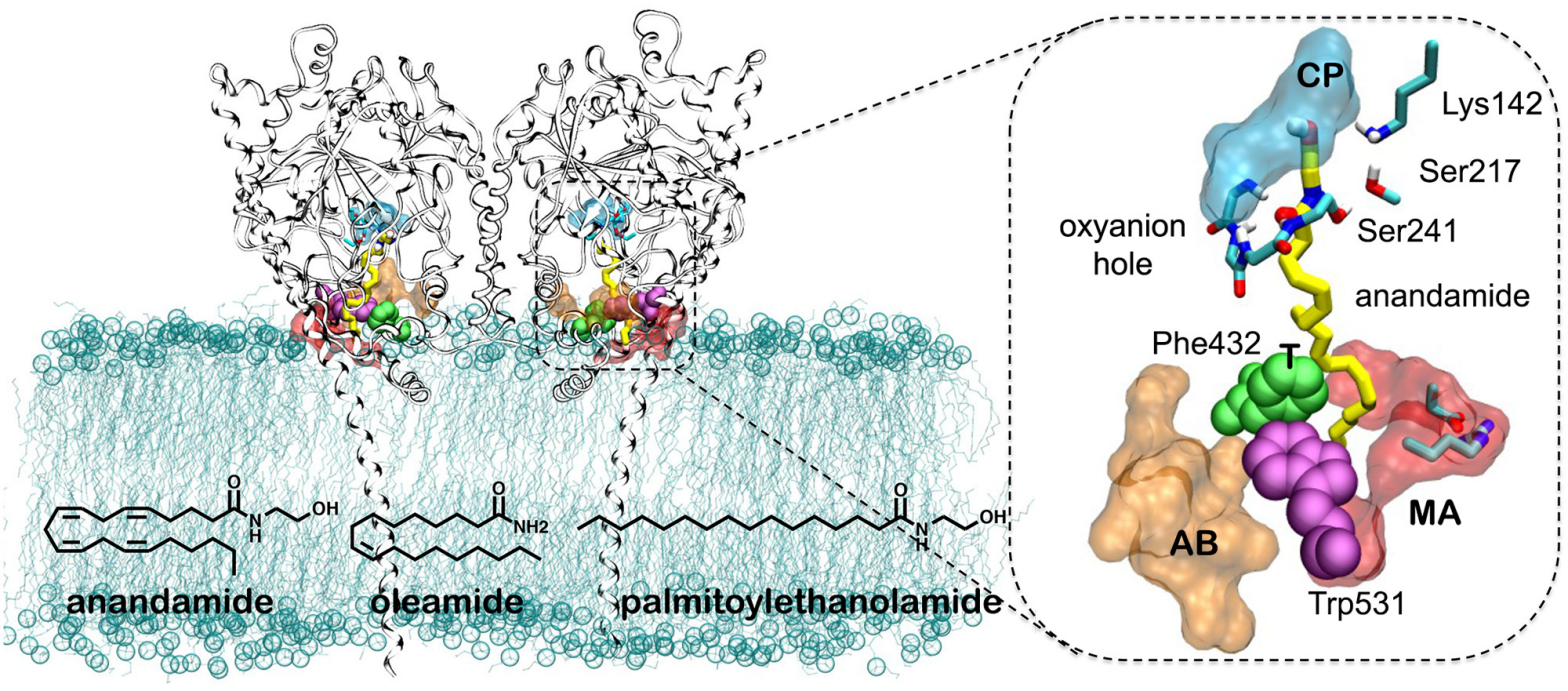
Overview of the FAAH protein (pdb 1MT5) [1] in complex with anandamide, embedded in a 1-palmitoyl-2-oleoyl-phosphatidylethanolamine (POPE) lipid bilayer. The enzyme is a homodimer, which is shown in gray ribbons. The lipids of the membrane are represented in cyan lines with the phosphate atoms highlighted as spheres. A close view of the binding site is shown on the right. The substrate anandamide is shown in yellow sticks, while the catalytic triad (Ser241-Ser217-Lys142) and the oxyanion hole (Ser241-Gly239-Gly240-Ser241) residues are represented in cyan sticks. The so called “membrane access” (MA—red) and the “acyl chain binding” (AB—orange) channels, as well as the “cytosolic port” (CP—cyan) are depicted in molecular surface representation. The interface region between the MA and AB channels is indicated as transition region (T). The Asp403 and Arg486 residues of the MA channel, which favor the substrates entrance within FAAH active site, are also shown as sticks. Key residues—Phe432 (green) and Trp531 (magenta)—are shown in space-filling representation. For clarity, explicit water molecules included in the simulations are omitted. The chemical structure of the FAAH substrates here considered—anandamide, oleamide, and palmitoylethanolamide (PEA)—is also shown.

Fatty acid amide hydrolase (FAAH—Fig 1) [1–3] and monoacylglycerol lipase (MAGL)[4] are two hydrolytic enzymes that mainly regulate the endocannabinoid system. These enzymes act on the endocannabinoid signaling mostly through hydrolysis of the endogenous substrates anandamide and 2-arachidonoylglycerol (2-AG), respectively.[5–7] Both FAAH and MAGL can also hydrolyze other lipids although less efficiently.[8–10] Regulation of the endocannabinoid system is a promising strategy for treating pain, cancer, and other inflammatory-related diseases, suggesting both FAAH and MAGL as effective drug targets.[11–18] It is therefore crucial to decipher the mechanisms for substrate selection and catalysis, which might help in the rational design of new therapeutics that act by modulating the endocannabinoid system. Building on our own and other relevant studies on FAAH catalysis and inhibition,[19–33] we provide here an elucidation of the main structural and kinetic features involved in substrate selection during FAAH catalysis.

Over the last decade, a wealth of experimental data has been generated on the structural properties and catalytic activity of FAAH.[5,7] FAAH is a homodimeric enzyme that can accommodate its substrate into a complex architecture of the catalytic site, which is characterized by three binding channels (Fig 1).[1] Substrates are thought to reach the catalytic site via a membrane access (MA) channel where two charged residues (Asp403 and Arg486) may favor the entrance of the polar head groups of fatty acid molecules. The catalytic action of FAAH occurs in the core of the binding site where an unusual catalytic triad (Ser241–Ser217–Lys142) performs the hydrolysis of the substrate, while an oxyanion hole (Ile238–Gly239–Gly240–Ser241) keeps the substrate properly oriented for hydrolysis. Tightly connected to the catalytic region, a cytosolic port (CP) allows the exit of the leaving group after substrate hydrolysis. A third acyl-chain binding (AB) cavity, adjacent to the MA channel, seems to contribute to the proper accommodation of the substrate during catalysis.[1,7]

The enzymatic activity of FAAH has been measured using different enzyme preparations and substrates,[9,10] showing that the enzyme displays a preferential hydrolytic activity for arachidonoyl substrates (20:4 (Δ^5,8,11,14^)), such as anandamide. Other substrates such as oleamide or palmitoylethanolamide, which contain a lower degree of unsaturation, are hydrolyzed at significantly slower and time-dependent rates (~50 to 100 times slower than anandamide after 5 minutes of incubation).[10] However, the structural and kinetic properties that regulate the preference of FAAH for its main substrate anandamide are still largely unknown. Based on structural data of FAAH, Mileni et al. originally proposed that the MA/AB boundary residue Phe432, in cooperation with the flexible residue Trp531, could act as a *dynamic paddle* that directs and orients the substrate during catalysis.[34] This mechanistic hypothesis was further corroborated by our recent computational study, which suggested that anandamide is not fully locked into the AB channel during catalysis, as previously supposed.[7,25] Rather, our study suggested that anandamide assumes hydrolysis-prone conformations by moving its flexible arachidonoyl chain between the MA and AB cavities interceded by the *dynamic paddle* residues that act as a gate between these two binding cavities.

To test this hypothesis and to elucidate the enzymatic strategy for substrate selectivity, we carried out long-timescale molecular dynamics (MD) simulations of FAAH embedded in a realistic membrane/water environment in complex with three substrates with different hydrolysis rates (anandamide > oleamide > palmitoylethanolamide) for both wild type and double mutant (Phe432Ala and Trp531Ala) systems. These unbiased microsecond MD simulations were accompanied by corresponding mutagenesis and kinetic experiments, which further validated the crucial role of Phe432 and Trp531 for substrate specificity. The integration of our theoretical and experimental results suggests indeed that lipid selection is attained through interplay of substrate and protein flexibility regulated by the *dynamic paddle*. In particular, the selective binding of specific lipid substrates seems to be regulated by the *dynamic paddle* residues that act as a gate between multiple binding pockets and that actively favor the formation of pre-reactive conformations for the preferred fatty acid substrate anandamide.

## Methods

### Structural models

We considered six model systems, each based on the X-ray structure of rat FAAH in complex with the anandamide analogue methyl arachidonoyl fluorophosphonate (MAFP), solved at 2.8 Å resolution (PDB code: 1MT5)[1]. As in our previous computational studies of FAAH,[24,25] these systems include the trans-membrane residues (9-29) and the N terminus, which were built by homology modeling. Three of these systems are formed by the wild type (*wt*) FAAH protein in complex with anandamide, oleamide, and palmitoylethanolamide (PEA) and are labeled *wt*FAAH/anandamide, *wt*FAAH/oleamide, and *wt*FAAH/PEA, respectively. The dynamic paddle residues (Phe432 and Trp531) were both mutated into alanine, obtaining the three corresponding double mutant systems (*mut*) *mut*FAAH/anandamide, *mut*FAAH/oleamide, and *mut*FAAH/PEA.

The initial binding mode of anandamide within the FAAH active site was taken from our previous studies.[24,25] Oleamide and PEA were docked using the Autodock 4.2 package.[35] The acyl chain of all three substrates was initially located in the MA channel, as suggested in refs.[1,7] Full details on the docking calculations are reported in the S1 Text.

The six FAAH/substrate systems were embedded into an explicit membrane environment formed by 480 1-palmitoyl-2-oleoyl-phosphatidylethanolamine (POPE) lipids.[1] Phosphatidylethanolamine is the major phospholipid of Escherichia coli membranes,[36] which was used as an expression system to produce purified proteins for the crystallization of the rat FAAH protein.[1] Each protein/membrane complex was hydrated with TIP3P[37] waters and 8 Cl^-^ counterions were added to neutralize the total charge. The size of the final systems was approximately ~145 Å x ~95 Å x ~140 Å, with ~35,500 water molecules and ~480 lipids, resulting in a total number of ~200,000 atoms for each system.

### Molecular dynamics simulations

The all-atom AMBER/parm99 force field was adopted for the FAAH protein. The anandamide, oleamide, and PEA lipids were treated with the General Amber Force Field (GAFF)[38] and the atomic charges were derived via the RESP fitting procedure.[39] Force field parameters for the lipid bilayer were taken from our previous studies on FAAH catalysis.[24,25] Force field parameters for the non-standard residues were carefully validated via electronic structure calculations, confirming the accuracy of the force field parameters used here.[25] The LINCS[40] algorithm was used to constrain covalent bonds involving hydrogens, allowing a time integration step of 2 fs. All the simulations were performed using GROMACS 4.[41] Long range electrostatic interactions were calculated with the particle mesh Ewald method with a real space cutoff of 10 Å. Periodic boundary conditions in the three directions of the Cartesian space were applied. The systems were coupled to a Nosé-Hoover thermostat[42,43] at a reference temperature of 310 K, and to an isotropic Parrinello-Rahman barostat[44] at a reference pressure of 1 bar both with a coupling time of 1 ps. The following simulation protocol was adopted: the systems were minimized using a steepest descent algorithm and then slowly heated up to 310 K in 1000 ps. This approach has been shown to be efficient for the equilibration phase of large biological systems (∼200,000 total atoms).[25,45,46] Under these conditions, POPE is a liquid–crystalline bilayer,[47,48] ensuring a realistic environment for the FAAH protein. Crucial membrane properties for the pre-equilibrated POPE membrane used here were carefully analyzed and have been published in our previous paper.[25] The simulations were performed with deprotonated Lys142, as proposed for the catalytic mechanism of FAAH.[1,49] Standard protonation states were maintained for the other protein residues. Approximately ~500–550 ns of MD simulations were collected in the NPT ensemble under standard conditions, for each of the six systems, resulting in a total of ~3–3.5 μs of dynamics. Coordinates of the systems were collected every 10 ps, for a total of ~50,000 frames for each run. Statistics were collected for the equilibrated systems after ~150 ns.

Binding free energies (Δ*G*_Bind_) for the three ligands in the *wt* and *mut* FAAH systems were estimated by the Molecular Mechanics/Poisson Boltzmann Surface Area (MM/PBSA)[50,51] approach implemented in the Amber 12 package.[52] Full details are given in the S1 Text.

Conformational and statistical analyses (see below) were performed over the equilibrated trajectories (last ~350 ns of MD) for all six simulations systems (~35,000 frames for each system). In all cases, both monomers yielded highly similar averages indicating that the system was well equilibrated (see S1 Text and S2-S3 Tables in S2 Text). Statistics were thus accumulated over both monomers resulting in an aggregate total sampling time of ~700 ns per system (~70,000 frames were considered for each system, with a total of ~420,000 analyzed frames). Data for each separate monomer of all the studied systems are also reported in S1 Text, S8 and S9 Figs, and S2 and S3 Tables in S2 Text.

### Analysis of molecular dynamics data

The root-mean-square-deviation (RMSD) after the equilibration time (~150 ns) was used as stability indicator, with respect to the crystal structure (S3–S6 Figs). The location of the substrates in either the MA or AB channel during the trajectories was identified by calculating the minimum distances *d* between the center of mass of the last three atoms of each substrates and the centers of mass of residues of the MA channel [(Asp403, Ile407, Arg486, Ile530)—*d-MA*], of the AB channel [(Tyr335, Glu373, Arg428, Phe527)—*d-AB*], and of the MA/AB transition region [(Phe381, Phe432, Trp531)—*d-T*], as in Palermo et al.[25] In detail, the substrates were located in MA if *d-MA* < 6 Å and *d-AB* > 6 Å; and in AB if *d-MA* > 6 Å and *d-AB* < 6 Å. If both these conditions were false and if *d-T* < 5 Å, the substrate’s acyl chain was considered to be located in the T region. The cutoff distances were chosen considering that the distance connecting the center of masses of the MA and AB channels is ~16/17 Å. Within this distance, ~6 Å each are occupied by the MA and AB channels, respectively (for a total of ~12 Å). The remaining ~4/5 Å therefore are considered as MA/AB interface region. The g-mindist tool of the GROMACS 4 package for MD analysis was used (S8 and S9 Figs). Full details on the substrate location are reported in S1 Text.

Conformational changes of the unsaturated lipids anandamide and oleamide were classified using the Applegate and Glomset notation.[53] Accordingly to the latter, unsaturated lipids assume different conformations that can be grouped in three major shapes: (i) *“elongated”; (ii) “hooked”*, and (iii) *“curved”*.[54,55] Due to the absence of double bonds in the palmitoyl chain of PEA, conformational changes were followed in this case by considering the change of the lipid length (end-to-end distance) with respect to the initial configuration. This allowed identification of *“elongated”*, *“hooked”*, and *“curved”* conformations too. Full details on the conformational analysis of the FAAH substrates considered in this study can be found in the S1 Text.

Conformational changes of the key Phe432 and Trp531 residues within the FAAH binding site were characterized by using the torsion angle *φ* along their Cα-Cβ axis, namely *φF* for Phe432 and *φW* for Trp531. The pre-organization of the FAAH active site to perform substrate hydrolysis was assessed via the definition of *catalytically significant conformational states* (for simplicity, here referred to as “pre-reactive states”) of the FAAH/substrate complex. These substrate conformations are those characterized by optimal distances and orientations of key structural parameters involved in the enzymatic reaction, as explained in detail in Palermo et al.[25] The structural parameters used here (S1 Text and S7 Fig) were identified based on several computational and crystallographic studies,[1,22,27,31,32,34,56] including our recent quantum mechanics/molecular mechanics (QM/MM) study of anandamide hydrolysis in FAAH.[24] It is important to mention that our definition of pre-reactive states only concerns the predisposition of the substrate to undergo hydrolysis, given the proper relative orientation of the substrate with respect to the catalytic residues in the binding pocket of FAAH. Finally, to analyze the role of the *dynamic paddle* in pre-reactive conformations, we report the trend of the *φF* and *φW* angles with respect to the location of the anandamide acyl chain in the MA, T, and AB regions, using polar coordinates.

### Lipid standards

Acetonitrile was purchased from Sigma Aldrich (Italy). High purity standard anandamide and PEA were purchased from Cayman Chemical (Ann Arbor, MI, USA).

### rFAAH cloning

The rat (r)FAAHΔTM (97-1722bp) cDNA was amplified by PCR from the cDNA clone 7370226 purchased from Open Biosystem (Thermo Scientific) using the following primer pair: forward 5’-GGGAATTCCATATGGGGCGCCAGAAGGCCC-3’; reverse 5’-ATAGTTTAGCGGCCGC**TCAATGATGATGATGATGATG**AGGGGTCATCAGCTG-3’ containing the NdeI and NotI restriction sites. A (6x)Histidine tag was introduced in the reverse primer sequence (bold). The amplified rFAAHΔTM was then cloned in pMALc5x vector in frame with the N-terminal MBP and finally introduced into *Escherichia coli* Rosetta gami 2 (DE3)-pLysS strain.

The F432A and W531A mutants were generated by site-directed mutagenesis using the QuickChange II Site-Directed Mutagenesis Kit (Agilent Technologies, Santa Clara, CA, USA) and the construct MBP-rFAAH-6xHis pMALc5x as template. The following primers were designed to introduce the single point-mutations Phe432Ala in rFAAH: forward 5’-CCTCGGCTGGCAGCCGCTCTCAACAGTATGCGTC-3’ and reverse 5’-GACGCATACTGTTGAGAGCGGCTGCCAGCCGAGG-3’; Trp531Ala in rFAAh forward 5’-GGCTACTTTGGGATATCGCGGACATCATCCTGAAG-3’ and reverse 5’-CTTCAGGATGATGTCCGCGATATCCCCAAAGTAGCC-3’.

### Protein expression and purification

Overexpression of the MBP-rFAAH-6xHis proteins was achieved in *E*. *coli* strain Rosetta gami 2 (DE3)pLysS (Novagen) by growing cells in LB medium at 37°C to an OD_600_ of 0.6, followed by induction with 0.25 mM isopropyl β-D-thiogalactopyranoside for 16 hours at 25°C. Cells were then harvested by centrifugation, resuspended in buffer [50 mM sodium phosphate pH 7.4, 0.2 M sodium chloride, 10 mM imidazole], and lysed by sonication. The lysate was incubated for 1h at 4°C with benzonase nuclease, 2 *μ*M MgCl_2_ and 1% Triton-X100. After centrifugation at 14,000 rpm for 30 min, the supernatant was incubated for 2 hours with NiNTA Agarose (Qiagen GmbH, Hilden, Germany) and washed with buffer containing increasing concentrations of imidazole (20 mM, 50 mM). Elution was performed with buffer containing 0.25 M imidazole. The buffer of the eluted sample was exchanged to 20 mM phosphate pH 7.4, 200 mM NaCl, 0.07% chaps.

### Enzyme kinetics experiments

Enzymes (*wt* and mutants) were dissolved in Tris-HCl 100 mM, pH 7.4 buffer and preincubated at 37°C for 10 minutes. Each substrate was then *individually* incubated at different concentrations (3.3, 6.25, 12.5, 25, 33, 50 *μ*M) with the enzymes. The highest substrate concentration was 50 μM and 33 *μ*M for anandamide and PEA, respectively, due to limited substrate solubility. The final enzyme concentration was kept at 10 nM. Reaction was stopped after 30 minutes at 37°C by addition of cold acetonitrile, assuming that a steady state was reached (Michaelis Menten condition). After mixing and centrifugation, an aliquot of the supernatant was used for UPLC-MS/MS analysis. Each experiment was run in triplicate. Enzyme velocity was calculated as pmoles of substrate consumed per minute per *μ*g of enzyme and plotted versus the concentration. Origin Pro 8.6 (OriginLab Corporation) was used to fit the velocity/concentration profiles and to determine the Michaelis Menten kinetic parameters (V_max_ and K_m_).

### Competition assay

Enzymes (*wt* and mutants) were dissolved at 10 nM concentration in Tris-HCl 100 mM, pH 7.4 buffer and preincubated at 37°C for 10 minutes. The reaction was then started by adding the substrates (anandamide and PEA) *simultaneously up* to a final 10 μM concentration. Final enzyme to substrate molar ratio was then 1 to 500. At different time points (0, 5, 15 and 30 minutes) an aliquot of the mixture was taken and the reaction was stopped by adding 4 volumes of cold acetonitrile. After mixing and centrifugation, an aliquot of the supernatant was used for UPLC-MS/MS analysis. Each experiment was run in triplicate. The incubation with rat liver microsomes (Tebu-Bio, Le Perray-en-Yvelines, France) was also prepared to run additional competition experiments (final concentration 0.1 mg/ml in the buffer).

### LC-MS analysis of anandamide and PEA

Anandamide and PEA levels were measured by LC-MS/MS on a Xevo-TQ triple quadrupole mass spectrometer coupled with a UPLC chromatographic system. Analytes were separated on a reversed phase BEH C18 column, using a linear gradient of acetonitrile in water. Column, UPLC, and MS were purchased from Waters Inc, (Milford USA). Quantification was performed monitoring the MRM transitions of the analytes. Analyte peak areas were compared with a standard calibration curve prepared in the 1 nM to 10 mM concentration range.

## Results

### MD simulations

Here, we considered five model systems together with the one reported recently in Palermo et al. (see Methods section).[25] Thus, the six model systems used for the comparative analysis are: wild type (*wt*) and mutated (*mut*) FAAH in complex with either anandamide, oleamide, or palmitoylethanolamide (PEA). The three *mut*FAAH systems are lacking the *dynamic paddle* residues (i.e. with both mutations Phe432Ala and Trp531Ala).

After equilibration (~150 ns for each system), FAAH is stable in all simulations, meaning that the backbone RMSD of the protein with respect to the initial crystallographic structure oscillates around 3 ± 0.1 Å for all six systems (see detailed data in SI). Interestingly, these extended simulations evidenced different conformations and locations of the three lipids within the FAAH active site, as induced by the presence/absence of the key Phe432/Trp531 *dynamic paddle*. In our analysis, these different substrate configurations are related to the propensity of FAAH to perform substrate hydrolysis, according to the definition of catalytically significant conformations (i.e. pre-reactive states of the FAAH/substrate complex—see the Methods section).[25]

### *wt*FAAH/anandamide

**Fig 2:**
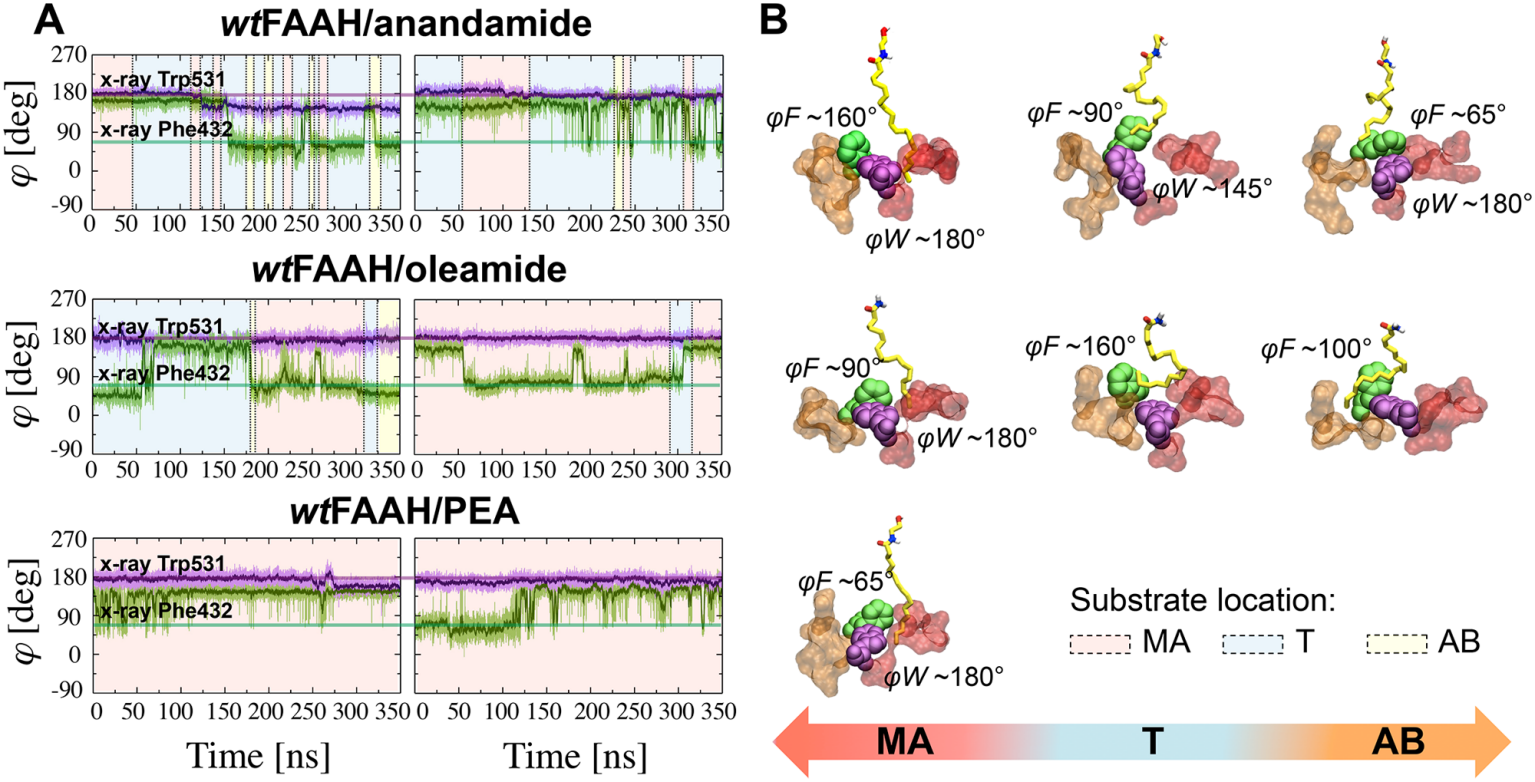
**(A)** Time evolution of the *φ* angle for Phe432 (*φF*—green) and Trp531 (*φW*—violet) in the *wt*FAAH/anandamide (first row), *wt*FAAH/oleamide (second row), and *wt*FAAH/PEA (third row) systems, shown for Monomer-A (first column) and Monomer-B (second column). Averages are shown in solid lines. Angles are expressed in degrees. The thick bars indicate the values of the *φ* angle for Phe432 (*φF* ~65°) and Trp531 (*φW* ~180°) in the X-ray structure (see Methods). The background colors indicate the location of the substrates acyl chain in the *wt*FAAH binding site. The background is in red for substrates located in the MA channel, in yellow when in AB, and in cyan at the MA/AB transition (T) region. **(B)** Selected snapshots from MD simulations showing the MA<–>AB transitions of the substrates are reported. The MA (red) and AB (orange) channels are shown in molecular surface, while FAAH substrates are shown in yellow sticks. Specific configurations of the Phe432 (green) and Trp531 (violet) that favor the location on the substrates in the MA, T, and AB regions are also shown (the *φ* angle is explicitly reported).

As discussed in detail in our previous study,[25] when initially located in the MA channel, anandamide reversibly transfers its arachidonoyl chain to the adjacent AB channel (Fig 2), without ever showing the arachidonoyl chain fully locked into the AB cavity.

**Fig 3:**
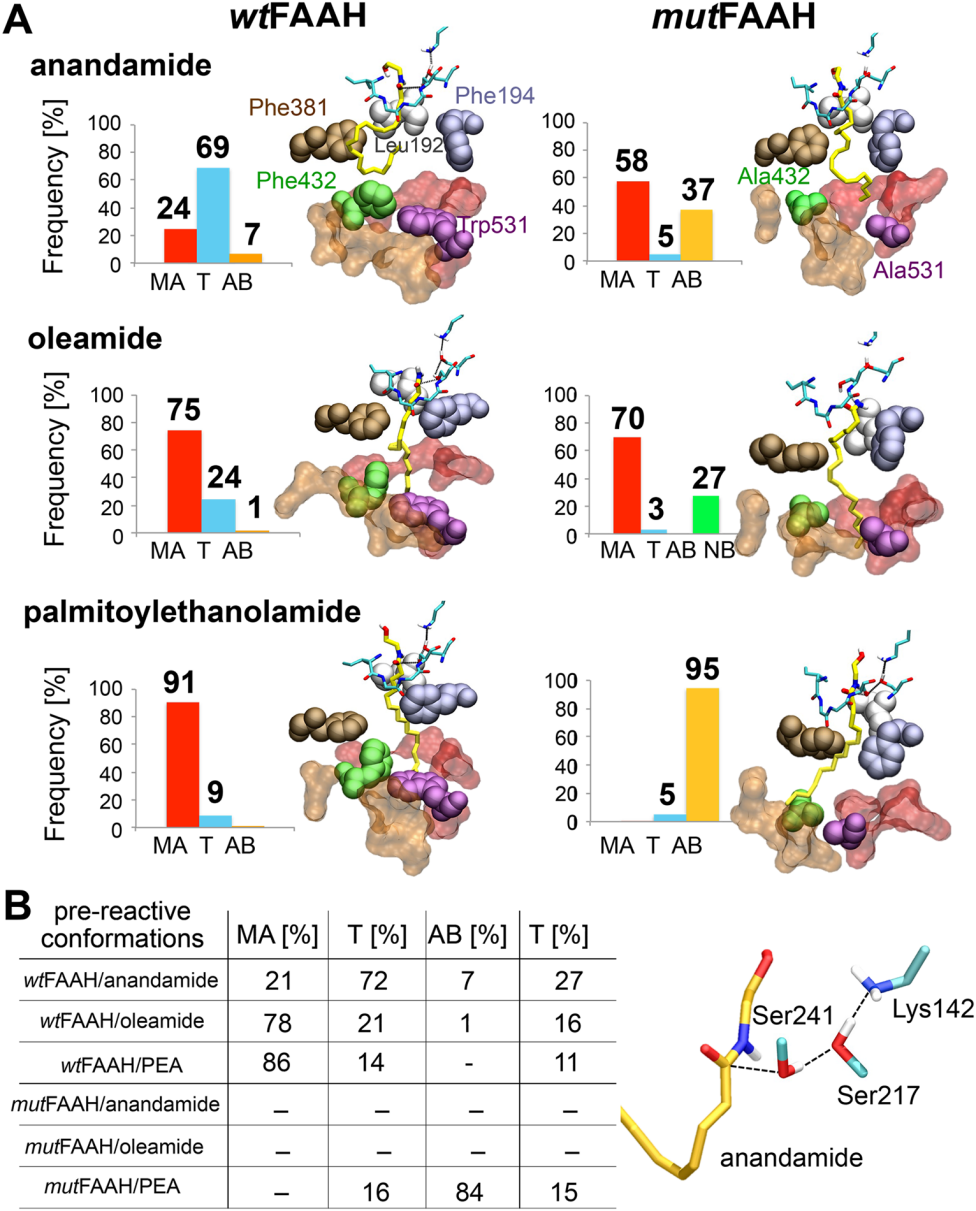
**(A)** Statistical distribution (% over the total equilibrated conformations—reported as bar graphs) of anandamide (first row), oleamide (second row), and palmitoylethanolamide (PEA—third row) conformations when located in the MA (red bars), T (cyan bars), and AB (orange bars) regions of the *wt*FAAH (first column) and *mut*FAAH (second column) systems. For the *mut*FAAH/oleamide system, the percentage of unbounded oleamide conformations (NB—not-bound) is also indicated with a green bar. For each system, one representative conformation of the most populated state is shown. The MA (red) and AB (orange) channels are shown in molecular surfaces. Key residues of the FAAH active site are in space-filling representation, namely: Leu192 (gray), Phe194 (ice blue), Phe381 (maroon), Phe432 (green), and Trp531 (violet). Phe432 and Trp531 are mutated in Ala in the *mut*FAAH systems. The substrates (yellow), as well as the catalytic triad and the oxyanion hole (cyan) are also shown in sticks. **(B)** Percentages of pre-reactive conformations of anandamide, oleamide, and palmitoylethanolamide (in rows) in the MA, T, and AB regions (in columns) of the *wt*FAAH and *mut*FAAH systems. The total percentage of pre-reactive conformations over the production runs is also reported in the last column. A representative picture of pre-reactive conformation is also reported (full details are reported in the S1 Text and in S7 Fig).

**Fig 4:**
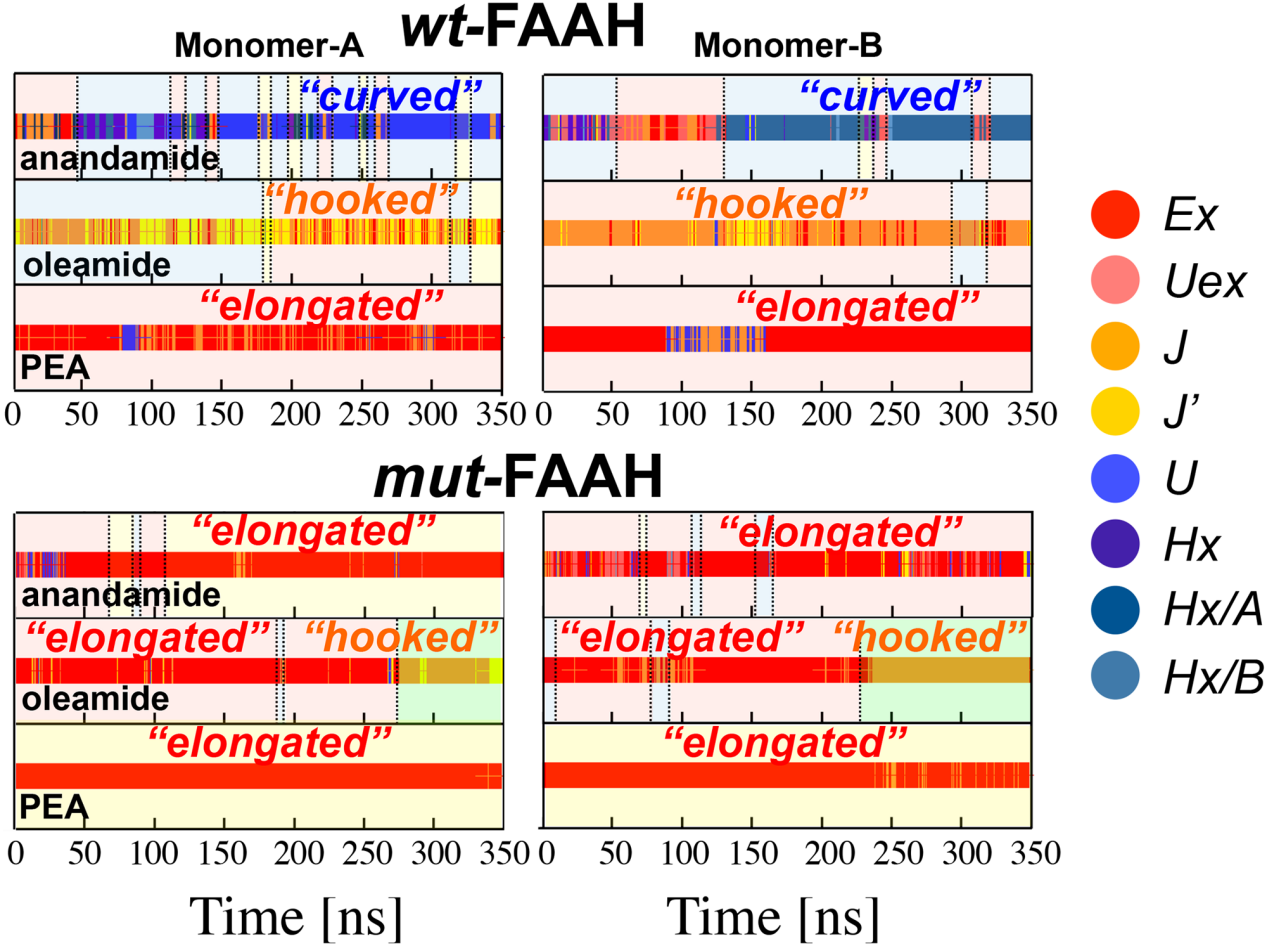
Time evolution of the *“elongated”* (red), *“hooked”* (yellow), and *“curved”* conformations of anandamide (first row), oleamide (second row), and PEA (third row), shown for Monomer-A (first column) and Monomer-B (second column) of the *wt*-FAAH (upper graphs) and *mut*-FAAH (lower graphs) systems. Time windows for the substrates location are shown with different background colors: red (MA channel), yellow (AB channel), and cyan (T region). The green background indicates the time windows for the oleamide unbinding in the *mut-*FAAH. Full details are in the main text, in the S1 Text and in S2 Fig.

After the equilibration time, 69% of the total anandamide configurations are located in the T region (where T stands for Transition region, which is located between the MA and AB pockets, Fig 3), while the population of the MA and AB channels is statistically less important (24% and 7%, respectively). Interestingly, pre-reactive conformations (27% of the production run) are mostly sampled while the lipid acyl chain is located in the T region (72%) and fewer conformations are located in the MA (21%) and AB (7%) channels. The anandamide’s tail preferentially assumes *“curved”* conformations due to the van der Waals interactions between its Δ ^8^/ Δ ^11^/ Δ^14^ double bonds and the aromatic rings of Phe432 and Phe381 (Figs 3 and 4).

**Fig 5:**
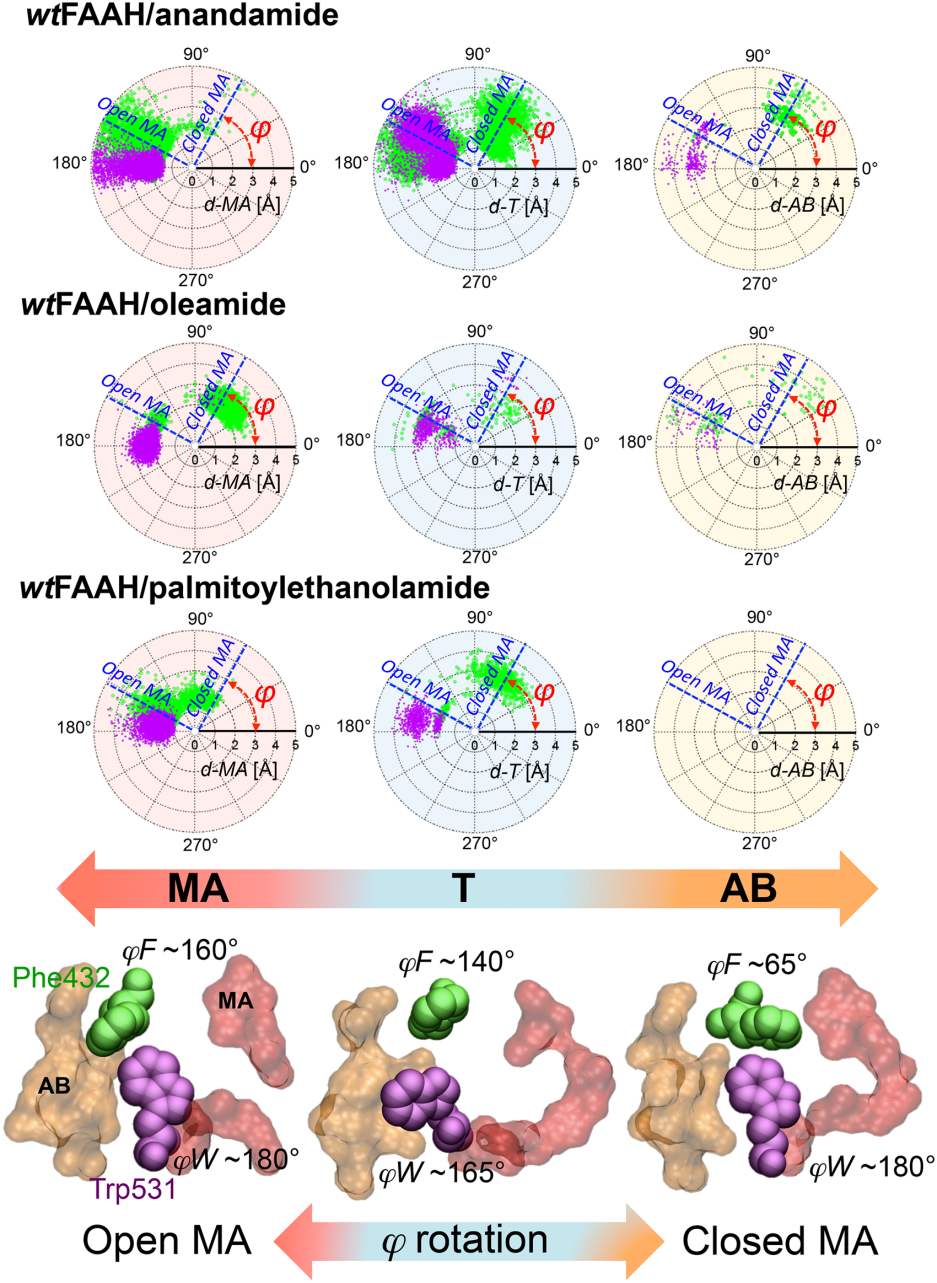
Polar plots of the *φ* angle (dihedral angle along the Cα-Cβ axis) of Phe432 (*φF*—green dots) and Trp531 (*φW*—violet dots) with respect to the *d-MA* (red background), *d-T* (cyan background), and *d-AB* (yellow background) distances for pre-reactive conformations of the *wt*FAAH/anandamide (first row), *wt*FAAH/oleamide (second row), and *wt*FAAH/palmitoylethanolamide (PEA—third row) systems. The polar (*d-MA*, *d-T* and *d-AB*) and angular (*φ—*in red on the plot) coordinates are explicitly indicated on the plots. The approximate values of *φF* of Phe432 for the *“open”* and *“closed”* MA channel configurations are highlighted with blue dashed bars. Distances and angles are expressed in Å and degrees, respectively. Definitions of the *d-MA*, *d-T* and *d-AB* distances are reported in the Methods section. Selected snapshots from MD simulations indicating the *“open”* (left) and *“closed”* (right) MA channel configurations, as induced by the rotation of the *φ* angle of Phe432 and the cooperative Trp531, are shown at the bottom of the polar plots. The MA (red) and AB (orange) channels are represented in molecular surfaces. Phe432 (green) and Trp531 (violet) are shown in space-filling representation. The *φ* angle of the two residues is explicitly reported.

Phe432 and Trp531 trigger the MA<–>AB transitions of anandamide, assuming different configurations that open and close the MA channel (Fig 2). This mechanism favors the proper location of pre-reactive conformations of anandamide between the two channels, as evidenced by the polar plot of the *φ* angles of Phe432 (*φF*) and Trp531 (*φW)* with respect to the location of the substrate in pre-reactive states (Fig 5). In detail, for pre-reactive conformations in the MA channel (red plot), the *φF* (green dots) ranges from ~120° to ~180° with the opening of MA. During the MA<–>AB transfer (cyan plot), the *φF* shows a bimodal distribution, given the rotation of *φF* from ~150° (*“open”* MA channel) to ~60° (*“closed”* MA channel, as observed in the X-ray structure), which permits the MA<–>AB transfer of the arachidonoyl chain. Trp531 contributes to this transfer, rotating *φW* by about ~35/40° (magenta dots). When pre-reactive conformations are in AB (yellow plot), Phe432 mainly closes the MA channel (*φF* ~65°), opening the adjacent AB channel, while Trp531 rotates *φW* from ~145° to ~180°.

### *mut*FAAH/anandamide

For the mutant form of FAAH, we detect several MA<–>AB transitions of the arachidonoyl chain (S9 Fig). After equilibration, the percentage of total anandamide conformations within the MA and AB channels is 58% and 37%, respectively, while only 5% of total anandamide conformations are found in the T region (Fig 3). This is primarily due to the absence of van der Waals interactions between the anandamide Δ^14^ double bond and Phe432 (in this system mutated to Ala), which instead are present in the *wt*-system. As a result, the arachidonoyl chain is mainly *“elongated”*, whereas it was mainly *“curved”* in *wt*FAAH (Fig 4). As a consequence, pre-reactive conformations are not sampled in the *mut*FAAH system, as anandamide never locates in the T region assuming the specific *“curved”* conformations that characterize the pre-reactive states in the *wt*-system (Figs 3 and 4).

### *wt*FAAH/oleamide

In this system, oleamide reversibly transfers its acyl chain from the MA to the AB channel, resembling the behavior observed for anandamide in the *wt*-system (Fig 2), although with less frequent transfers. In both enzymatic subunits, Phe432 shows several dihedral transitions, thus assuming two different configurations that lead to the “open” (*φF* ~160°) and “closed” (*φF* ~65°—X-ray) MA channel configurations, whereas Trp531 does not undergo any conformational transitions.

After equilibration, the percentage of oleamide conformations within the MA channel is 75%, while the T region and the AB channel are poorly populated (24% and 1%, respectively—Fig 3). Pre-reactive conformations are sampled for 16% of the total equilibrated trajectory, therefore much less than for anandamide. The 78% of these conformations are located in the MA channel, 21% in the T region, and only a few are sampled in the AB channel (1%—Fig 3). The different preferential location for oleamide in MA, compared to anandamide, can be explained by the presence of only one double bond (Δ^9^) of the oleoyl chain, which results in weaker interactions with the aromatic residues Phe381/Phe432 (Fig 3). This also explains the formation of *“hooked”* configurations of the oleoyl chain (Fig 4).

When oleamide is in a pre-reactive state and located in MA, *φF* shows a bimodal distribution (red plot in Fig 5B—*φF* angle is shown in green dots), therefore opening (*φF* ~180°) and closing (*φF* ~60°) the MA channel. This bimodal behavior of the *dynamic paddle* mechanism (which is similarly detected for pre-reactive conformations in T and AB) allows the proper location of the shorter oleoyl chain in the MA channel, via the formation of van der Waals interactions with the Δ^9^ double bond of the lipid and Phe432 (Fig 3).

### *mut*FAAH/oleamide

During the simulations, oleamide never transfers its acyl chain from the MA channel into the adjacent AB channel (S9 Fig). This explains that 70% of the total oleamide configurations are located in the MA channel, while only a few conformations of the lipid are located in the T region (3%) and no conformations are detected in the AB channel (Fig 3). When located within the *mut*FAAH active site, oleamide is preferentially *“elongated”*, as the oleoyl acyl chain is not bent by Phe432 (Fig 4). As in the *mut*FAAH/anandamide system, pre-reactive conformations are not sampled for oleamide in complex with the *mut*FAAH protein. This highlights the crucial role of the Phe432/Trp531 gating residues in inducing specific conformations for hydrolysis of these lipids.

Interestingly, oleamide spontaneously unbinds from the FAAH active site and locates within the lipid bilayer for 27% of the overall production run. Oleamide unbinding occurs in both FAAH subunits (at ~425 ns in mnr-A and at ~350 ns in mnr-B) and via the same mechanism. As previously suggested,[1,7,25] two charged residues (Asp403–Arg486) facilitate the passage of the substrate through the MA channel, H-bonding to the polar head group of the substrate (S10 Fig). Surrounded by lipids, oleamide mainly assumes *“hooked”* conformations (Fig 4), in agreement with the proposal that FAAH substrates need to adopt a closed *“hairpin-like”* conformation to be transported across the membranes.[57,58] The mutation of Phe432, which in the *wt*-system interacts with the oleamide Δ^9^ double bond, causes a destabilization of the oleoyl chain within the active site. In addition, oleamide is a primary amide that, therefore, does not have the ability to form H-bond interactions with the CP residue Thr236, which is critical for leaving group departure, after substrate hydrolysis.[1,7,25] A detailed description of oleamide unbinding in the *mut*FAAH protein is reported in S1 Text and S10 Fig.

### *wt*FAAH/palmitoylethanolamide (PEA)

The lipid remains mainly located in the MA channel in both the enzyme subunits, for the whole simulation time (Fig 2C) and no MA<->AB transfers of the PEA acyl chain occur. Phe432 assumes different conformations via the rotation of *φF* from ~65° to ~160°, allowing an optimal fit of the long palmitoyl chain into the MA channel. Trp531 does not undergo dihedral transitions during the simulation, further stabilizing the palmitoyl chain in the MA channel.

After the equilibration time, 91% of the total PEA configurations are located in the MA channel and 9% locate in the T region (Fig 3). Pre-reactive conformations are observed for 11% of the production run. Most of these conformations are located in the MA channel (86%), whereas fewer conformations locate in the T region (14%, Fig 3), and none in AB. The palmitoyl tail preferentially assumes *“elongated”* shapes (Fig 4), given the absence of unsaturation within the lipid. This prevents PEA from establishing specific interactions with Phe381/Phe432 and catalyzing the MA<–>AB switch, which is similar to the process observed with oleamide and anandamide. Therefore, the lipid acyl chain does not undergo any bending, which explains the fully elongated shapes and the absence of MA<–>AB transfers.

### *mut*FAAH/PEA

Here, PEA transfers its palmitoyl chain from the MA to the AB channel in the early phase of the equilibration (~10/20 ns), in both FAAH monomers (S9 Fig). After equilibration, conformations of PEA detected within the MA channel are statistically irrelevant (0%), whereas 5% of PEA conformations are located in the T region and 95% within the AB channel (Fig 3). The lipid remains anchored to the end of the AB channel throughout the simulations, strongly interacting with Tyr225 and Phe527. Meanwhile, the head of the substrate is H-bonding with Thr236 at the top of the active site. These interactions favor the formation of *“elongated”* conformations (Fig 4).

Pre-reactive conformations are sampled for 15% over the whole production run. Most of the pre-reactive conformations are sampled when the palmitoyl acyl chain is located in the AB channel (84%) and in the T region (16%). These conformations are not sampled in the MA channel (Fig 3B).

### Enzyme kinetic experiments

To further characterize the proposed mechanism for substrate selection during FAAH catalysis, we expressed and purified the recombinant *wt* rat FAAH protein (MBP-rFAAH-6xHis construct) and we also introduced a single point mutation for each of the two paddle residues (Phe432Ala and Trp531Ala). The activity of the purified *wt* and two mutant proteins was tested using enzyme kinetic experiments in the presence of different concentrations of each substrate (anandamide and PEA), as reported in the method section. The enzymatic reactions were quenched after 30 minutes when a steady-state equilibrium was reached (i.e., Michaelis Menten condition).

The K_m_ value obtained for the *wt* protein in the presence of the anandamide substrate was equal to 5.26 *μ*M, in excellent agreement with the results reported by Labar G. et al. (K_m_ = 5.31 *μ*M)[59] for the recombinant MBP-FAAH construct. The overlay of the kinetic curves obtained for anandamide shows that the enzyme velocity (pmol of substrate consumed per minute per *μ*g of protein) is only slightly higher for the *wt* protein compared to the two mutant proteins (Table 1). Thus, neither of the two mutations seems to significantly affect the affinity of FAAH for the anandamide substrate under steady-state conditions. With the PEA substrate, we find an enzyme affinity that is 2-fold lower then the one for anandamide, with a K_m_ equal to 12.53 *μ*M for the *wt* protein. Here too, each of the two mutations only marginally affects the affinity of the enzyme for the PEA substrate (K_m_ values in Table 1). Overall, steady-state conditions confirm that FAAH has better affinity for anandamide, over PEA, for wtFAAH.[9]

**Table 1:** K_m_ obtained from the fitting of the kinetic assays (S12 Fig).

anandamide	K_m_
WT	5.26
F432A	5.80
W531A	6.15
**PEA**	**K**_**m**_
WT	12.53
F432A	14.04
W531A	16.10

y = V_max_*x/(K_m_+x) (Origin Pro 8.6)

### Competition assays

We further performed competition experiments under non-equilibrium conditions, which is often reported to be the case for biochemical reactions *in vivo*.[60–62] These are performed on a mixture of the three proteins, i.e. the *wt*FAAH and the two Phe432Ala and Trp531Ala mutants, in the presence of both anandamide and PEA. The enzymatic reaction was quenched at different time points, within the initial 30 minutes of reaction, and the products were then analyzed by UPLC-MS/MS (see Methods section).

**Fig 6:**
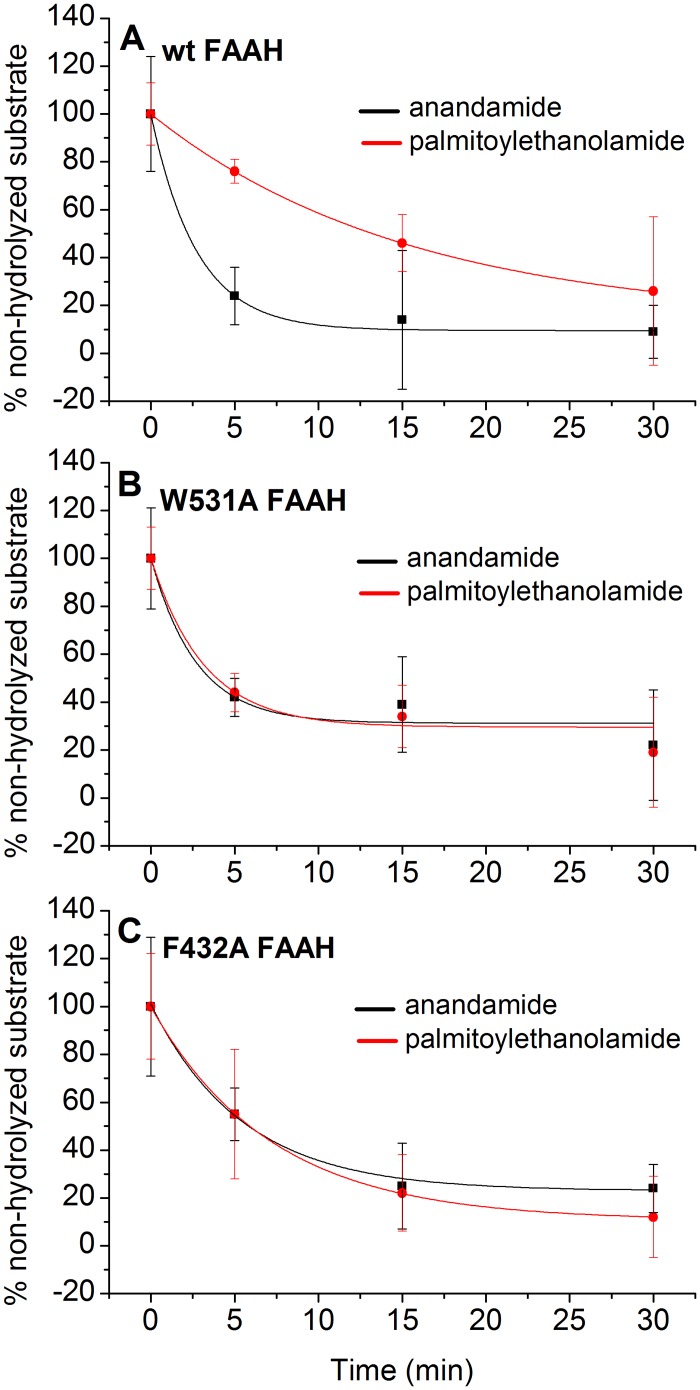
Competition assays performed for the *wt* (A), W531A (B), and F432A (C) proteins in the presence of both anandamide (black) and PEA (red). Each set of data was fitted using simple exponential decay functions, whose parameters are reported in S6 Table in S2 Text.

As reported in Fig 6, for the *wt*FAAH recombinant protein, the rate of anandamide hydrolysis is 5.6 times faster than for PEA (S6 Table in S2 Text). Single point mutants (Phe432Ala and Trp531Ala) showed similar decay rates for both anandamide and PEA substrates (Fig 6 and S6 Table in S2 Text).

We also tested the validity of our competition assay, described in the Methods section, on a rat liver microsomes preparation. As expected, we found that FAAH preferentially cleaves its main substrate, anandamide, rather than PEA (S11 Fig; S7 Table in S2 Text) in rat liver microsomes. This is in agreement with Desarnaud et al.,[10] who reported that the FAAH hydrolytic function is more efficient for anandamide than for PEA in rat brain microsomes. A consistently faster rate of hydrolysis for anandamide, compared to PEA, was observed in both the recombinant form of FAAH and in rat liver microsomes. This suggests that the microsomal membrane may not critically affect substrate selectivity in FAAH catalysis.

## Discussion

Extensive classical molecular dynamics simulations have been coupled with mutagenesis and kinetic experiments to understand the enzymatic mechanism for substrate selectivity of FAAH, which hydrolyzes a variety of lipids with however different catalytic rates. We correlated the dynamics of FAAH with its experimental activity, when in complex with anandamide, the primary substrate of FAAH, and two less efficiently hydrolyzed substrates, oleamide and palmitoylethanolamide (PEA). The *dynamic paddle* residues Phe432 and Trp531 have been hypothesized to regulate both selectivity and activity of FAAH.[7,25] To better dissect their specific role, we considered wild-type (*wt*) and mutated Phe432Ala and Trp531Ala systems, both in MD and in single-point mutagenesis experiments.

During the MD simulations of the *wt*FAAH systems, we detected a different preferential location of the three substrates (anandamide, oleamide, and PEA) within the enzymatic active site. Importantly, the exact location of the substrate is strictly related to the presence/absence of double bonds within the lipid chain (Figs 2 and 3). Along the MD runs, the substrate population in the transition (T) region, which indicates MA<–>AB transfers, increases according to the number of double bonds located in the acyl chain of the substrate. Anandamide (4 *C = C*) locates in the T region for 69% of the simulation time, while only 24% of oleamide conformations and 9% of PEA conformations are sampled in the T region. This indicates that the four unsaturations in anandamide form favorable van der Waals interactions with the gating residue Phe432, explaining why anandamide is mostly located in the T region. This hydrophobic interaction is less pronounced with oleamide (one *C = C*), and not possible with PEA (no *C = C*).

In the T region, both anandamide and oleamide undergo a conformational change that favors their interaction with the gating Phe432 residue. As a result, the acyl chain of those unsaturated substrates is bent in the T region, in the *wt*FAAH active site (Fig 4). In this way, the substrate is properly located for hydrolysis, forming pre-reactive conformations, i.e. conformations more prone to undergoing hydrolysis. Therefore, the presence of unsaturations seems a pre-requisite for efficient substrate hydrolysis.[25] Importantly, this would explain why the rate for substrate hydrolysis increases with the number of double bonds of the substrate lipid chain, as observed in previously published experimental data.[9,10] The time-dependent hydrolysis of linolenoyl (3 *C = C*) substrates is 45:100 times slower than anandamide, while eicosadienoyl (2 *C = C*), oleoyl (1 *C = C*) and the palmitoyl chains (no *C = C*) substrates are hydrolyzed, respectively, at rates 35:100, 2:100, and 1:100 times slower than anandamide.[10] Also, Boger et al. have shown that the incorporation of π-unsaturations at the arachidonoyl Δ^8,9^/Δ^1,12^ and oleoyl Δ^9,10^ locations into several α-ketoheterocycles inhibitors greatly enhances the potency of those compounds,[63] suggesting that bent conformations of the ligand are essential for tight binding to FAAH.[9,64] Anandamide forms the highest percentage of pre-reactive conformations compared to the other substrates. In fact, pre-reactive conformations are differentially sampled by each substrate (*wtFAAH/anandamide* 27%, *wtFAAH/*oleamide 16%, and *wtFAAH/*PEA 11%). Binding free energies (*Δ*G_Bind_—see Methods section, S1 Text and S4 Table in S2 Text), obtained via the MM/PBSA method,^49,50^ confirm the highest affinity of anandamide for the *wt*FAAH. Oleamide’s affinity for FAAH is ~5 kcal mol^-1^ lower than that found for anandamide. PEA shows ~11 kcal mol^-1^ lower affinity for FAAH, compared to anandamide. This further suggests that the van der Waals interaction between the substrate unsaturations of anandamide and the Phe432 side chain at the T region can favor binding and catalysis.

MD simulations show that in the *mut*FAAH double mutant, anandamide preferentially locates in the MA channel. Due to the absence of the key interface Phe432/Trp531 residues, and rarely locates itself in the T region (Fig 3). As a result, pre-reactive conformations are not sampled in the *mut*FAAH/anandamide, which is similar to observations for the *mut*FAAH/oleamide. Notably, in *wt*FAAH/PEA and *mut*FAAH/PEA, pre-reactive conformations are observed for 11% and 15% of the production runs, respectively. These data indicate that the presence/absence of the *dynamic paddle* does not significantly affect the formation of PEA pre-reactive conformations in FAAH. In the case of the unsaturated lipids—i.e., anandamide and oleamide—the presence of the dynamic paddle is crucial in determining the formation of catalytically competent states of the complex. In fact, pre-reactive conformations are not observed in the *mut*FAAH/anandamide and *mut*FAAH/oleamide systems. These data further suggest the dynamic paddle as crucial in selecting the pre-reactive states for the hydrolysis of the unsaturated lipids in FAAH[9, 10]. Moreover, *“elongated”* conformations of PEA are sampled in both the *wt* and *mut* FAAH systems (Fig 4). The lack of unsaturations in the substrate’s tail explains the similar behavior of PEA in *wt* and *mut* systems, further corroborating the specificity of the *paddle residues* for unsaturated lipids only, such as anandamide. Taken together, these results indicate that, in the absence of the *dynamic paddle*, or in the absence of unsaturations in the substrate’s tail, the ligand does not assume *“curved”* (anandamide) and *“hooked”* (oleamide) conformations at the T region in the catalytic pocket. Notably, these conformations characterize the pre-reactive states in *wt*FAAH,[25] indicating that *curved* and *hooked* conformations are probably catalytically relevant for FAAH-mediated hydrolysis.

Mutagenesis and enzyme kinetic experiments, performed using anandamide and PEA, further confirm the importance of the *dynamic paddle* mechanism for substrate selection in FAAH, especially using competition assays in non-equilibrium conditions. In fact, in *wt*FAAH, the K_m_ for anandamide is 2-fold higher than for PEA (Table 1).[9,10] In steady-state conditions, however, the single mutations of the two paddle residues did not significantly affect the enzyme kinetic parameters. Nevertheless, when we performed competition assays in non-equilibrium conditions,[10] we observed that FAAH had a larger affinity for anandamide than for PEA. In this case, the rate of hydrolysis for anandamide was 5.6 times faster than for PEA after 30 mins, measured for the *wt*FAAH recombinant protein (S6 Table in S2 Text), while much higher in the very first 5 mins (see Fig 6A), as observer in Desarnaud et al.[10] When single point mutations (Phe432Ala or Trp531Ala) were inserted, results showed similar decay rates for both anandamide and PEA substrates, further suggesting the paddle mechanism as a key to substrate selectivity.

The experimental single point mutations were also able to discern the contribution to the paddle mechanism of each one of the two key residues Phe432 and Trp531. In fact, the mutations Phe432Ala and Trp531Ala show that the two residues Phe432 and Trp531 affect the paddling mechanism differently. The Trp531Ala mutation preserves the decay rate of anandamide, as observed in the *wt* protein, while the Phe432Ala mutant induces a hydrolysis rate of anandamide that is two times slower than the *wt* protein (S6 Table in S2 Text). This suggests a predominant role of the gating Phe432 residue in the paddle mechanism for lipid selection and substrate hydrolysis. This agrees with our MD simulations, which indicate Phe432 as the main player in the formation of specific *“curved”* pre-reactive conformations of anandamide (Fig 4). Phe432 primarily mediates the mutual opening and closure of the MA and AB channels through a gating mechanism, allowing a proper location of the unsaturated chains for hydrolysis (Fig 5), while Trp531 mostly exerts a cooperative role in this process.[65] PEA is also hydrolyzed twice as fast by Trp531Ala and more than four times as fast by Phe432Ala when compared to the *wt*, further suggesting that the paddle mechanism is only selective for anandamide (Fig 6). This is in very good agreement with our computational findings. As reported above, we detect a higher occurrence of pre-reactive conformations of PEA in *mut*FAAH (15%) with respect to *wt*FAAH (11%), while the calculated binding free energy (*Δ*G_Bind_) of PEA to *mut*FAAH is ~7 kcal mol^-1^ higher than the one for *wt*FAAH (see S1 Text and S4 Table in S2 Text).[50,51]

Interestingly, it has recently been suggested that the membrane itself may have a role in supporting substrate entrance towards the catalytic site,[65] somehow helping substrate selectivity. In this respect, we also performed kinetics experiments in rat liver microsomes, confirming the evidence obtained by Desarnaud et al.[10] in rat brain microsomes, with FAAH preferentially hydrolyzing its main substrate anandamide, compared to PEA (S11 Fig; S7 Table in S2 Text). These findings are in agreement with our experiments on substrate selectivity performed using the recombinant *in vitro* protein in the absence of membranes. We used a transmembrane domain-deleted FAAH construct, which was still catalytically active and able to bind the membranes, as reported by Patricelli et al.[66] The substrate selectivity, observed in the microsomes, is similar to that observed for the recombinant form. This suggests that the membrane’s contribution is not likely to significantly affect the mechanism for substrate selectivity in FAAH.

**Fig 7:**
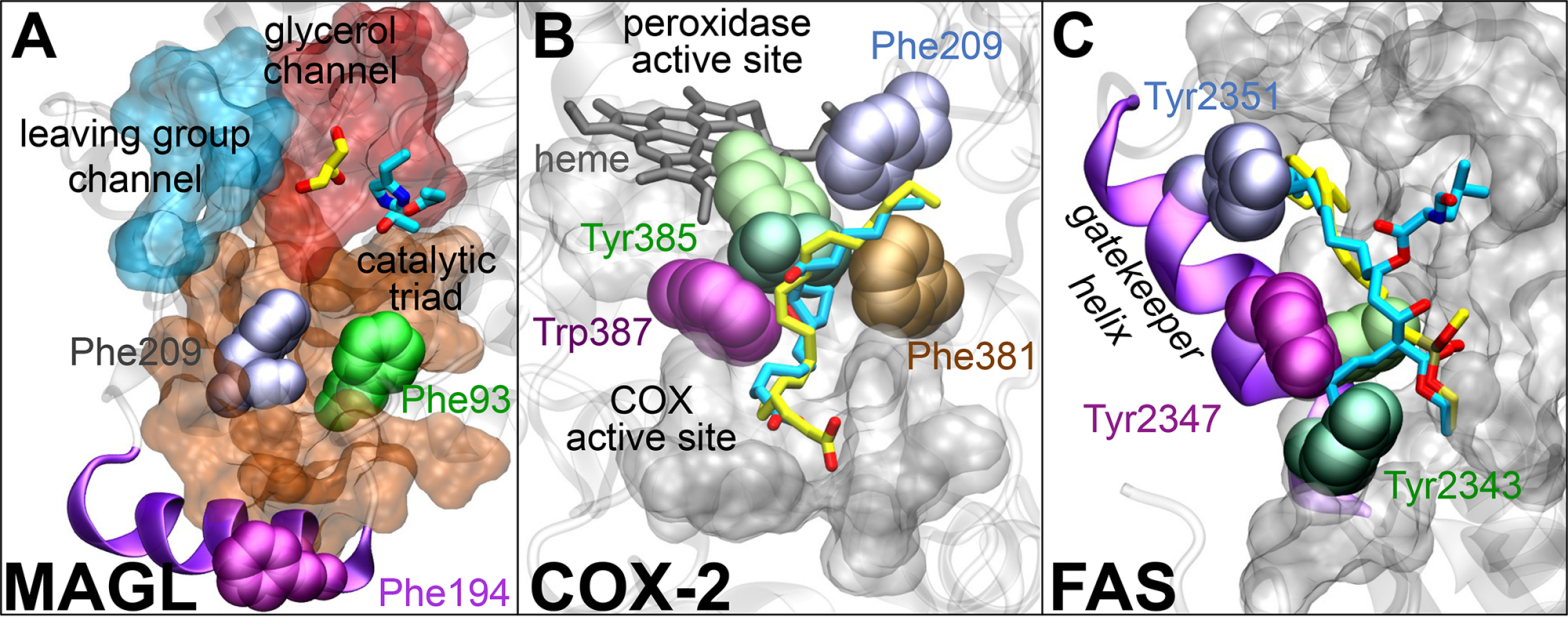
**(A)** Active site of the *apo* form of monoacylglycerol lipase (MAGL –3HJU.pdb).[4] A long acyl-chain channel (orange) is supposed to accommodate the arachidonoyl chain of 2-arachidonoylglycerol (2-AG), thanks to the presence of the aromatic Phe93 (green) and Phe209 (magenta). At the top of the active site, a polar cavity (red) likely accommodates the glycerol head group of 2-AG, as a glycerol molecule (yellow sticks) has been crystallized in this region. A third cavity (cyan) is thought to allow the exit of the substrate leaving group, after hydrolysis. The Ser122–Asp239–His269 catalytic triad is also shown with cyan sticks. **(B)** Superimposition of the cyclooxygenase COX-2 in complex with the arachidonic acid substrate (yellow— 1DIJ.pdb) and with the prostaglandin H_2_ (PGH_2_) product (cyan— 1DDX.pdb).[71] The catalytic Tyr385 is in dark green when in the presence of arachidonic acid and light green when in the presence of PGH_2_. It shows a conformational change that opens the gate from the cyclooxygenase to the peroxidase active site. For clarity, the key Trp387 (magenta), Phe381 (maroon), and Phe209 (ice blue) are only shown for the 1DIY.pdb. The *heme* co-factor is also shown. The cyclooxygenase and peroxidase active sites are in gray molecular surface. **(C)** Active site of the human fatty acid synthase (FAS) covalently bound to the methyl γ-linolenylphosphonate substrate (yellow— 3TJM.pdb)[75] and to the antitumor drug Orlistat (cyan— 2PX6.pdb).[76] In the presence of the γ-linolenyl chain, Tyr2343 (light green) forms a *“gatekeeper helix”* with Tyr2351 (magenta) and Tyr2347 (sky blue), leading to the formation of a long groove tunnel for the selective binding of the linolenyl chain. When bound to Orlistat, Tyr2351 (dark green) is buried within the active site, causing the loss of the *“gatekeeper helix”* mechanism, which is not formed. Tyr2351 (magenta) and Tyr2347 (ice blue), as well as the *“gatekeeper helix”* (violet ribbons), are only shown for the 3YJM.pdb. The protein active site is in gray molecular surface.

The idea that the selective binding of long and flexible lipid substrates to lipid-processing enzymes could be facilitated by the presence of multiple pockets in one catalytic site, as described here, is also supported by several other pieces of experimental data. Crystallographic and biochemical studies have suggested that FAAH could adapt to the chemical nature of different lipid substrates or inhibitors thanks to a pronounced flexibility of its binding channels.[34,56,65,67–70] A mix of hydrophobic/hydrophilic pockets, similar to that found in FAAH, was also found in the active site of another endocannabinoid enzyme MAGL, which primarily hydrolyzes the endocannabinoind 2-arachidonoylglycerol (2-AG, Fig 7A).[4] MAGL’s catalytic site shows a long and bipartite acyl-chain channel that faces the membrane, likely allowing the entrance of the flexible lipid chain of 2-AG from the lipid bilayer. This channel hosts two Phe residues (i.e., 93 and 209), which, similarly to the *dynamic paddle* mechanism observed in FAAH, could assist the binding of the arachidonoyl chain within the catalytic site during catalysis. Indeed, docking studies showed that 2-AG might differentially locate its arachidonoyl chain with respect to Phe93 and Phe209, thus suggesting a possible gating role of those residues in selecting specific conformations of 2-AG into the catalytic pockets.[4] An additional polar cavity is located at the top of the active site and likely accommodates the glycerol head group of 2-AG for subsequent hydrolysis.[4,8] Finally, a third binding cavity in MAGL, an opening with a diameter of ~5 Å connects the active site to the outside of the protein, ensuring the leaving group departure, similarly to the CP cavity in FAAH.

It is interesting to consider the catalytic site architecture of other lipid-degrading enzymes that show structural features, similar to FAAH, which might be required for substrate selection. The first examples are the cyclooxygenase COX-1 and COX-2 enzymes, which specifically transform the product of FAAH catalysis (the arachidonic acid) into prostaglandin H_2_ (PGH_2_) via two sequential reactions that occur in two spatially distinct active sites (Fig 7).[71,72] Interestingly, X-ray structures of COXs show that the interaction between the arachidonoyl tail of the substrate and the aromatic Tyr385, Trp387, Phe209, and Phe381 residues leads to specific conformations of the arachidonic acid, which sensibly differ from those of other fatty acids that are processed with lower catalytic efficiency.[71–73] The human fatty acid synthase (FAS) is a further example of a lipid-processing enzyme that shows flexible binding channels and gating residues. FAS is responsible for the *de novo* biosynthesis of long-chain fatty acids.[74] In this case, high-resolution crystal structures show that the covalently bound *γ*-linolenyl substrate selectively binds in a long groove tunnel site. It has been suggested that this makes the selection for the C_18_ substrates (Fig 7).[75] The comparison between the active site region of FAS covalently bound to either the *γ*-linolenyl substrate[75] (C_18_ and 3 *C = C*) or to the antitumor drug Orlistat[76] (characterized by the saturated palmitic chain) shows a conformational change of Tyr2343, again suggesting a possible gating mechanism for lipid selection. Upon *γ*-linolenyl substrate binding, Tyr2343 forms a *“gatekeeper helix”* with Tyr2351 and Tyr2347 that is not observed in the presence of Orlistat. Finally, multiple channels in a single active site are also reported in other lipid-degrading enzymes, such as lipase hydrolyzing triglycerides, which show varying substrate specificity for different lipid acyl chains. This seems to depend on the shape and length of the fatty acid binding cavity.[77],[78]

Overall, these data suggest that structural flexibility, gating residues, and multiple binding pockets are key to lipid selection in FAAH catalysis and, probably, in other structurally similar lipid-degrading enzymes. The concomitant existence of these structural features seems crucial to facilitating the preferential binding of the selected lipid within a broad spectrum of endogenous molecules. In FAAH, the interplay between the ligand and protein structural flexibility seems crucial to understanding the lipid selection mechanism, as mediated by key gating residues that form the *dynamic paddle*. Finally, we have shown that this structural framework for lipid selection could probably be further extended to several other lipid-processing enzymes.

### Conclusions

Here, long time-scale classical molecular dynamics simulations have been integrated with mutagenesis and kinetic experiments in order to clarify the molecular basis for substrate selectivity in FAAH catalysis. Extensive MD simulations of FAAH in complex with its main substrate anandamide have been compared with simulations where FAAH is in complex with less efficiently hydrolyzed substrates (oleamide and palmitoylethanolamide). This comparative study has revealed that FAAH selectively accommodates anandamide into a multi-pocket binding site, and properly orients it in pre-reactive conformations for efficient hydrolysis. Mutagenesis and kinetic experiments have further highlighted the importance of Phe432 and Trp531 for substrate selection in competition assays in non-equilibrium conditions. The interplay between ligand and protein structural flexibility seems crucial for lipid selection during catalysis in FAAH, as mediated by the gating residues Phe432 and Trp531 that form the *dynamic paddle*, which facilitates the formation of pre-reactive conformations of the substrate/enzyme complex.

Based on existing structural data, we propose that our results could be extended to other lipid-processing enzymes where the presence of multiple binding cavities and gating residues have been indicated to be relevant for enzyme selectivity and function. One example is MAGL, another endocannabinoid enzyme, which primarily hydrolyzes 2-arachidonoylglycerol. A broader validation of this structural framework for lipid selection, with additional experimental and/or theoretical investigations, would be very informative and applicable to de-novo enzyme design and drug discovery efforts.[79]

## Supporting Information

S1 Fig Initial conformation of the studied substrates anandamide (A), oleamide (B) and PEA (C) within the *wt*FAAH active site (PDB code: 1MT5). Residues belonging to the MA (red) and AB (orange) channels are shown in molecular surfaces. Phe432 (green) and Trp531 (violet) are shown in space-filling representation. The substrates (yellow) and the Ser241-Ser217-Lys142 catalytic triad (cyan) are shown as sticks.
(TIF)

S2 Fig Chemical structure (top) and conformations (bottom) of anandamide (A), oleamide (B) and PEA (C). Anandamide conformations are classified in three classes: *(i)* class of the *“elongated”* shapes that include the extended *Ex* and extended U *Uex* shapes; *(ii)* class of the *“hooked”* shapes including the *J* and *J’* shapes; *(iii)* class of the *“curved”* shapes comprising the *U*, helical *Hx* and the half helical *Hx-A/B* shapes. Oleamide conformations are classified as *“elongated”* (*Ex*), *“hooked”* (*J/J’*) and *“curved”* (*Hx*), as well. PEA conformations are “*extended” Ex*, *“hooked” J* and “*helical” Hx* conformations. The lipids are depicted in yellow sticks. Torsion angles ω used to define the lipid conformations are highlighted in red, blue, and green colors. Details are reported in S1 Text.
(TIF)

S3 Fig RMSD of the protein along MD simulations of the *wt*FAAH systems. Time evolution of the RMSD for the backbone atoms of the overall FAAH protein (blue), the crystallographic residues [30–597 (red)] and the modeled trans membrane residues [1–29 (green)], shown for the *wt*FAAH/anandamide (upper graph), the *wt*FAAH/oleamide (central graph) and the *wt*FAAH/PEA (lower graph) systems. The gray background indicates the equilibration time (~150 ns).
(TIF)

S4 Fig RMSD of the protein along MD simulations of the *mut*FAAH systems. Color code as S3 Fig.
(TIF)

S5 Fig RMSD of the ligands along MD simulations of the *wt*FAAH systems. Time evolution of the RMSD for the heavy atoms of the anandamide, oleamide and PEA substrates in monomer-A (blue) and monomer-B (red) of the *wt*FAAH/anandamide (upper graph), the *wt*FAAH/oleamide (central graph) and the *wt*FAAH/PEA (lower graph) systems. Averages are shown in corresponding solid lines. The gray background indicates the equilibration time (~150 ns).
(TIF)

S6 Fig RMSD of the ligands along MD simulations of the *mut*FAAH systems. Color code as S5 Fig.
(TIF)

S7 Fig Definition of catalytically significant conformations. Distances **(A)** and angles **(B)** used to define the catalytically significant conformations in the FAAH/substrates complexes, shown for the anandamide substrate.
(TIF)

S8 Fig Location of the substrates acyl chain in the *wt*FAAH systems. Time evolution of the minimum distances between the last three atom of the substrates acyl chain and the residues belonging to the MA channel (red), AB channel (orange) and to the MA/AB interface region [i.e., T region (blue)], shown for monomer-A (Mnr-A, first column) and monomer-B (Mnr-B, second column) of the *wt*FAAH/anandamide (upper graph), the *wt*FAAH/oleamide (central graph) and the *wt*FAAH/PEA (lower graph) systems. Averages are shown in corresponding solid lines. The g-mindist tool present in the GROMACS 4 package for MD analysis was used. The gray background indicates the equilibration time of the systems. Time windows for the location of the substrates are indicated with different color backgrounds: red (MA channel), yellow (AB channel) and cyan (MA/AB interface).
(TIF)

S9 Fig Location of the substrates acyl chain in the *mut*FAAH systems. Color code as S8 Fig.
(TIF)

S10 Fig Unbinding mechanism of oleamide in the *mut*FAAH systems. In the *mut*FAAH/oleamide system, oleamide spontaneously unbinds from the FAAH active site using the MA channel as an exit route. The unbinding mechanism is favored by two charged residues of the MA channel (Asp403 and Arg486, which are shown in cyan sticks) that facilitate the passage of the substrate through the MA channel, H-bonding to the polar head group of the substrate. The oleamide unbinding occurs in both FAAH monomers (at ~425 ns in mnr-A and at ~350 ns in mnr-B). The MA (red) and AB (orange) channels are depicted in molecular surface representation. The mutated *“dynamic paddle”* residues Ala432 (green) and Ala531 (green) are shown in space-filling representation. The enzymatic framework is shown in gray ribbons, while the lipids of the membrane are represented in cyan lines. Water molecules accessing the MA channel are also shown as sticks and balls.
(TIF)

S11 Fig Competition assays performed for a rat liver microsome preparation in the presence of both substrates anandamide (black) and PEA (red). The reactions were quenched at different time points with the addition of four volumes of cold acetonitrile. Each set of data was fitted using simple exponential decay functions, whose parameters are reported in S7 Table in S2 Text.
(TIF)

S12 Fig Kinetic experiments showing the rate of hydrolysis of the anandamide and PEA substrates for the *wt* enzyme (black), the F432A mutant (red), and the W531A mutant (blue). Reaction was stopped after 30 minutes at 37°C assuming that a steady state was reached (Michaelis Menten condition). Each set of data was fitted using a Michaelis Menten model. Details are reported in the main text.
(TIF)

S13 Fig Graphical representation of the main message of this work. (TIF)

S1 Text Supporting Text section. Full docking details. Details on the conformational analysis of the lipid substrates. Definition of catalytically competent conformational states of the FAAH/substrate complexes. Reproducibility of the results in both FAAH subunits. Details on the location of the substrates within FAAH active site during MD. Details on the occurrence of pre-reactive conformational states during MD. Unbinding of oleamide in the *mut*FAAH system. Binding free energy calculations.
(DOCX)

S2 Text Supporting Information including S1–S7 Tables. (DOCX) Supporting tables including docking data (S1 Table), statistical analysis of the occurrence of catalytically relevant conformations (S2-S3 Tables), binding free energies (Δ*G*_Bind_—S4 Table) and Δ*G*_Bind_ energetic contributions (S5 Table). Supporting data about competition assays are also reported (S6-S7 Tables).
(DOCX)
