# Supplementary figures and images for: Keys to Lipid Selection in Fatty Acid Amide Hydrolase Catalysis: Structural Flexibility, Gating Residues and Multiple Binding Pockets
Source: PLoS Comput Biol. 2015 Jun 25;11(6):e1004231. doi: 10.1371/journal.pcbi.1004231 (PMC4481349; doi:10.1371/journal.pcbi.1004231)

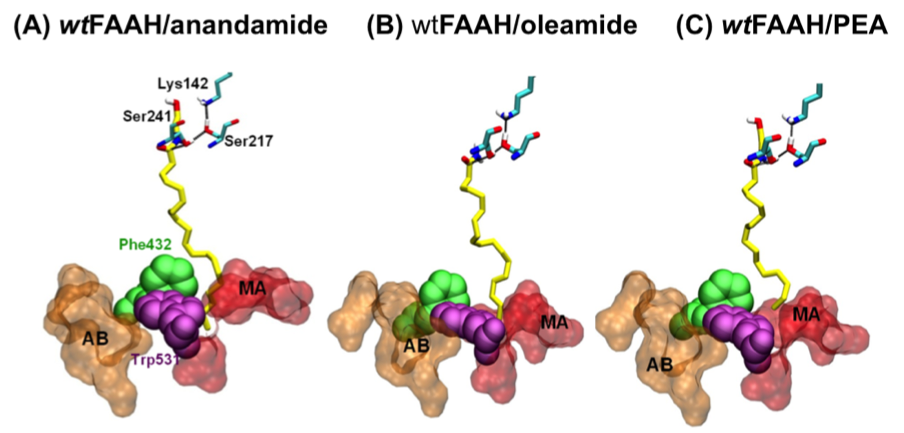

Supplement: S1 Fig — Residues belonging to the MA (red) and AB (orange) channels are shown in molecular surfaces. Phe432 (green) and Trp531 (violet) are shown in space-filling representation. The substrates (yellow) and the Ser241-Ser217-Lys142 catalytic triad (cyan) are shown as sticks. (TIF) [file pcbi.1004231.s001.tif]

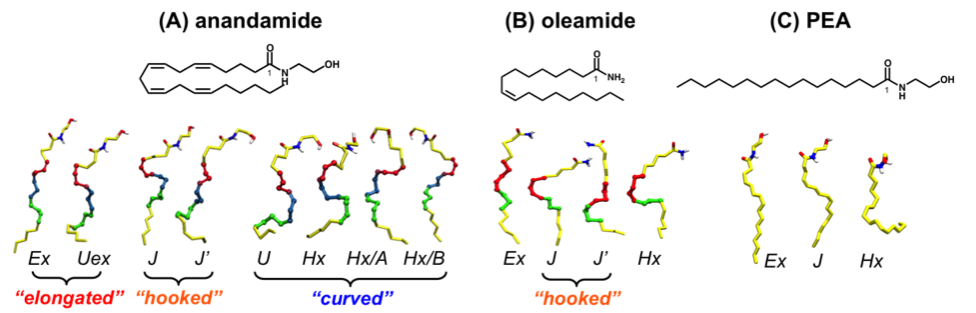

Supplement: S2 Fig — Anandamide conformations are classified in three classes: (i) class of the “elongated” shapes that include the extended Ex and extended U Uex shapes; (ii) class of the “hooked” shapes including the J and J’ shapes; (iii) class of the “curved” shapes comprising the U, helical Hx and the half helical Hx-A/B shapes. Oleamide conformations are classified as “elongated” (Ex), “hooked” (J/J’) and “curved” (Hx), as well. PEA conformations are “extended” Ex, “hooked” J and “helical” Hx conformations. The lipids are depicted in yellow sticks. Torsion angles ω used to define the lipid conformations are highlighted in red, blue, and green colors. Details are reported in S1 Text. (TIF) [file pcbi.1004231.s002.tif]

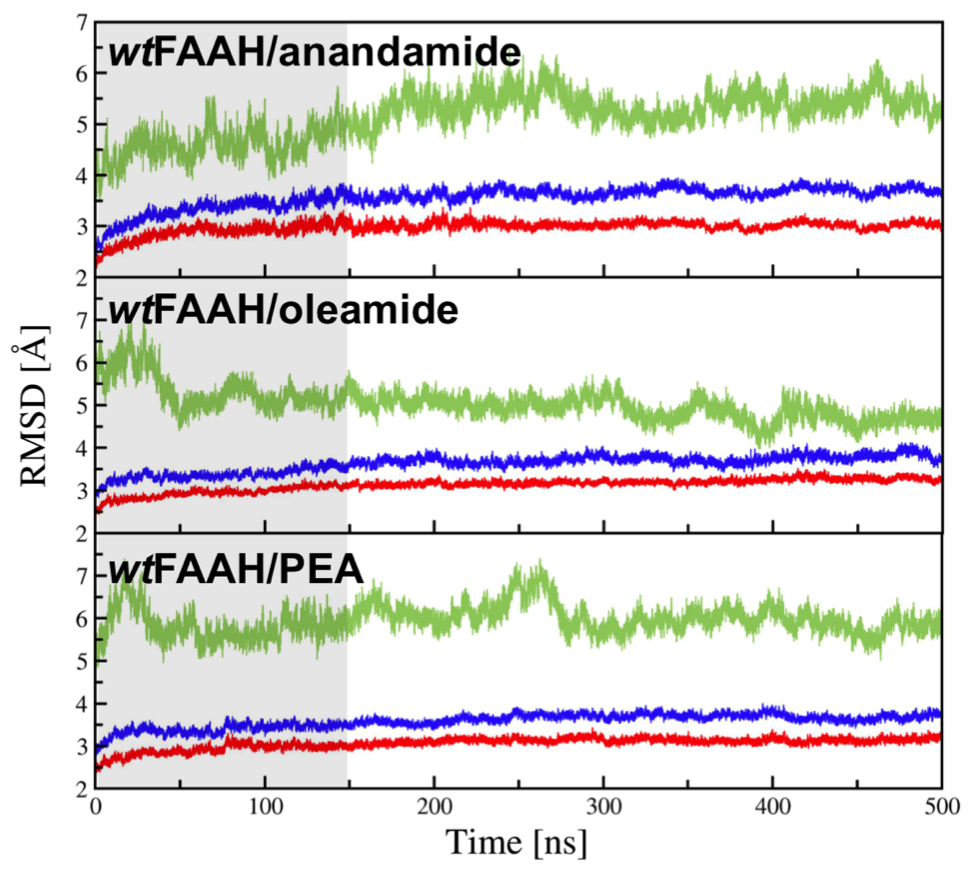

Supplement: S3 Fig — Time evolution of the RMSD for the backbone atoms of the overall FAAH protein (blue), the crystallographic residues [30–597 (red)] and the modeled trans membrane residues [1–29 (green)], shown for the wtFAAH/anandamide (upper graph), the wtFAAH/oleamide (central graph) and the wtFAAH/PEA (lower graph) systems. The gray background indicates the equilibration time (~150 ns). (TIF) [file pcbi.1004231.s003.tif]

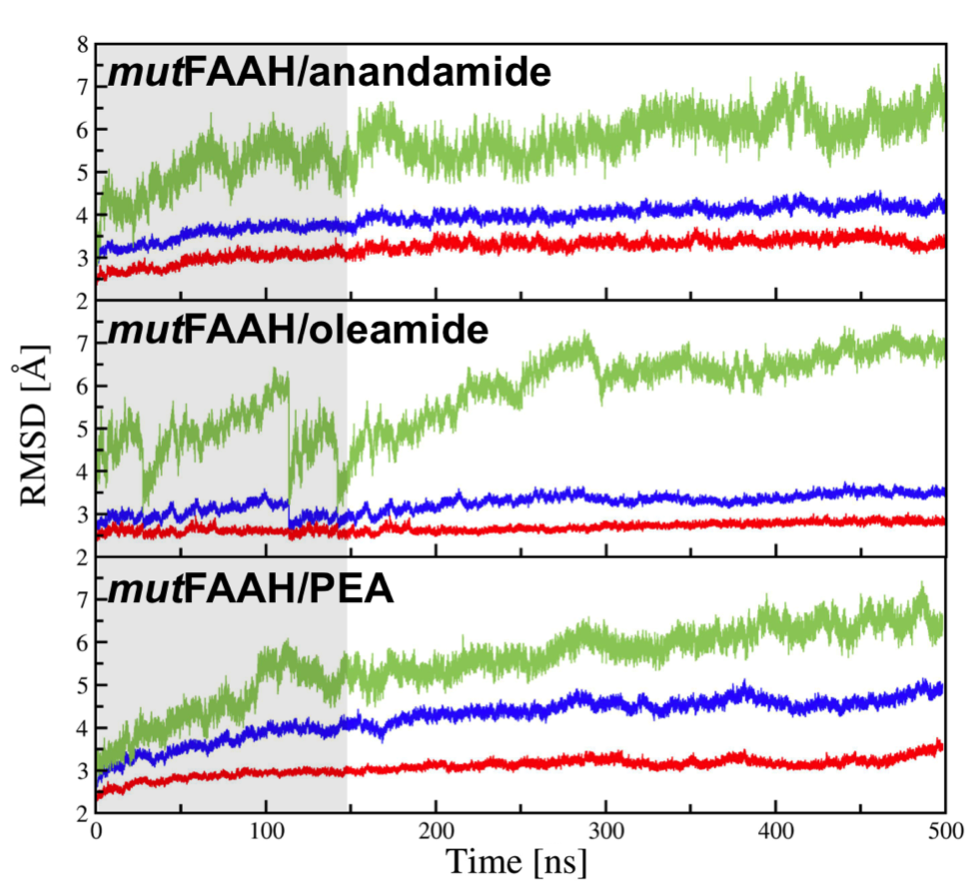

Supplement: S4 Fig — Color code as S3 Fig. (TIF) [file pcbi.1004231.s004.tif]

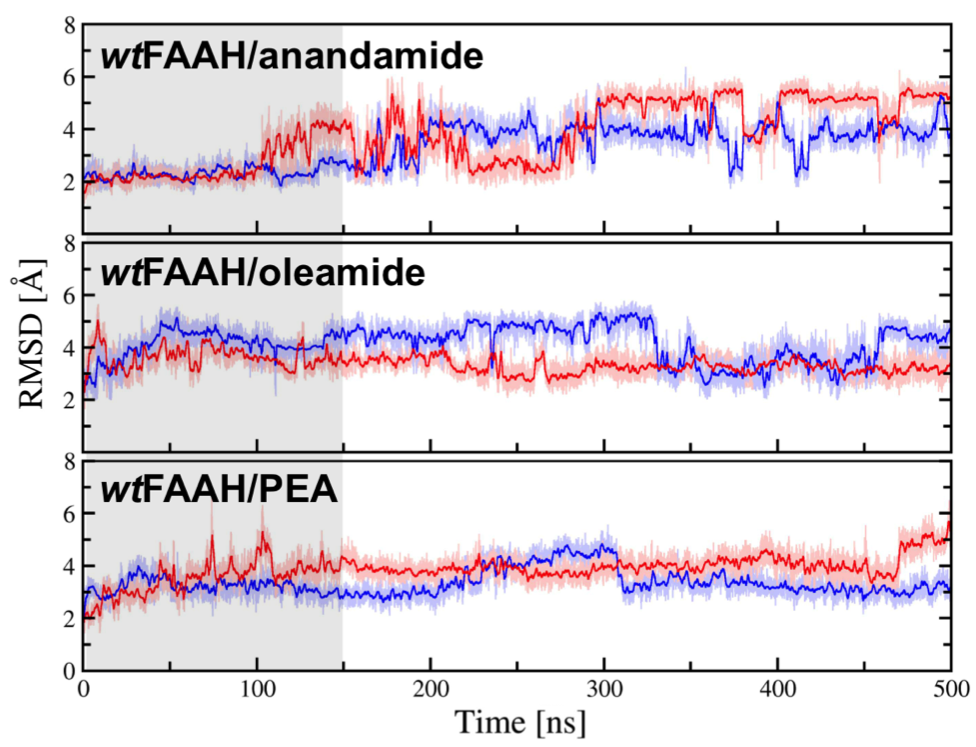

Supplement: S5 Fig — Time evolution of the RMSD for the heavy atoms of the anandamide, oleamide and PEA substrates in monomer-A (blue) and monomer-B (red) of the wtFAAH/anandamide (upper graph), the wtFAAH/oleamide (central graph) and the wtFAAH/PEA (lower graph) systems. Averages are shown in corresponding solid lines. The gray background indicates the equilibration time (~150 ns). (TIF) [file pcbi.1004231.s005.tif]

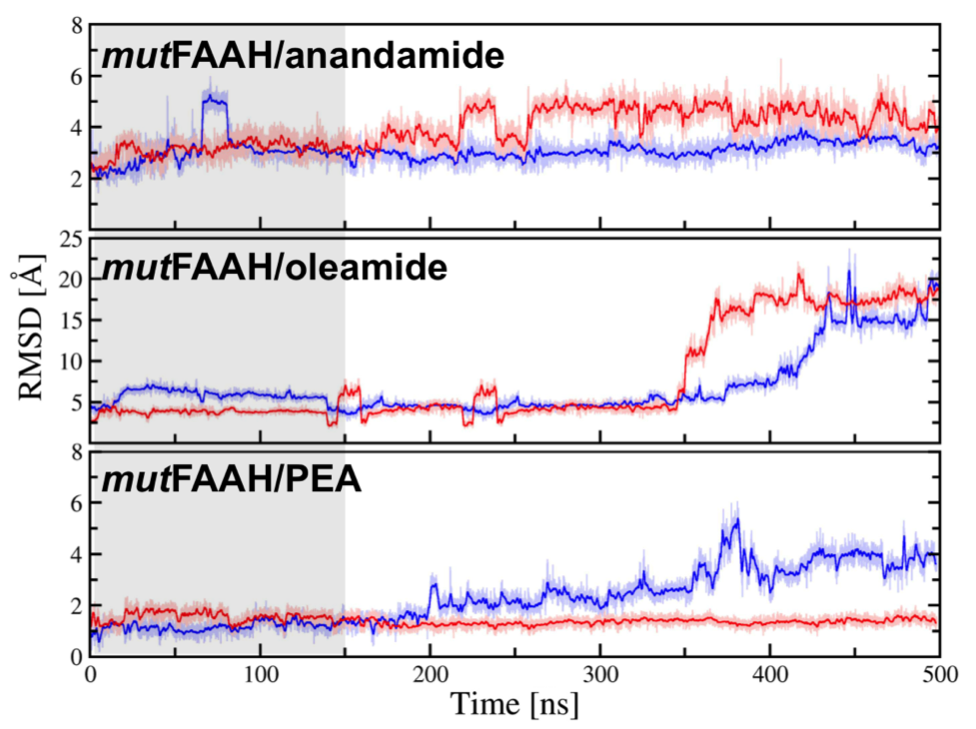

Supplement: S6 Fig — Color code as S5 Fig. (TIF) [file pcbi.1004231.s006.tif]

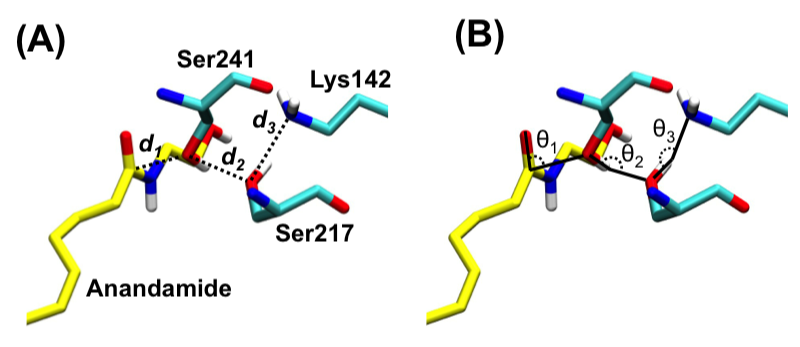

Supplement: S7 Fig — Distances (A) and angles (B) used to define the catalytically significant conformations in the FAAH/substrates complexes, shown for the anandamide substrate. (TIF) [file pcbi.1004231.s007.tif]

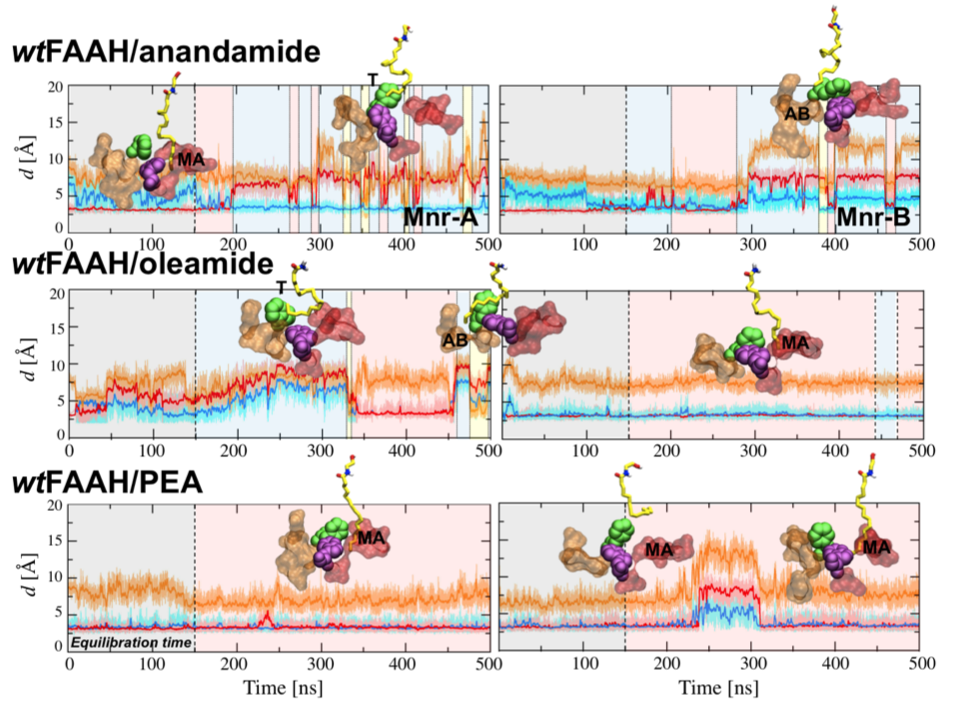

Supplement: S8 Fig — Time evolution of the minimum distances between the last three atom of the substrates acyl chain and the residues belonging to the MA channel (red), AB channel (orange) and to the MA/AB interface region [i.e., T region (blue)], shown for monomer-A (Mnr-A, first column) and monomer-B (Mnr-B, second column) of the wtFAAH/anandamide (upper graph), the wtFAAH/oleamide (central graph) and the wtFAAH/PEA (lower graph) systems. Averages are shown in corresponding solid lines. The g-mindist tool present in the GROMACS 4 package for MD analysis was used. The gray background indicates the equilibration time of the systems. Time windows for the location of the substrates are indicated with different color backgrounds: red (MA channel), yellow (AB channel) and cyan (MA/AB interface). (TIF) [file pcbi.1004231.s008.tif]

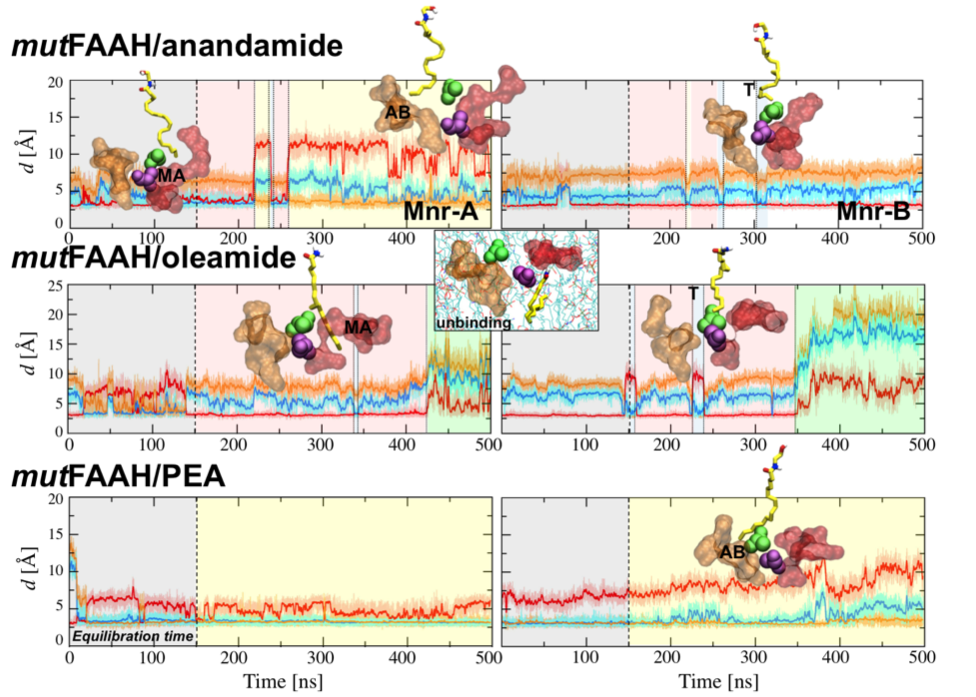

Supplement: S9 Fig — Color code as S8 Fig. (TIF) [file pcbi.1004231.s009.tif]

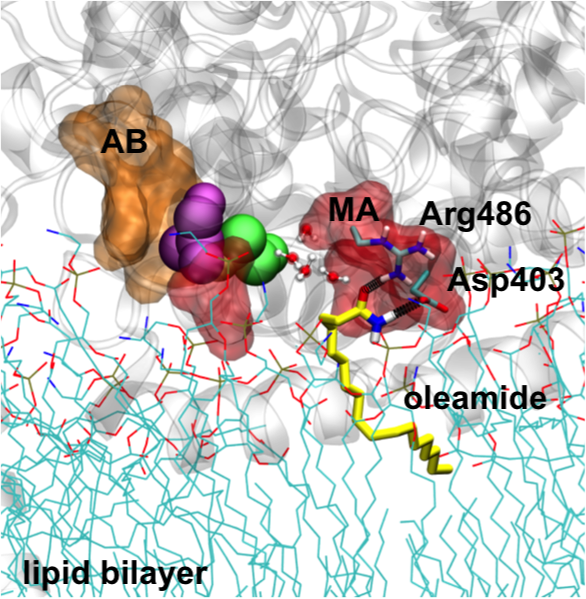

Supplement: S10 Fig — In the mutFAAH/oleamide system, oleamide spontaneously unbinds from the FAAH active site using the MA channel as an exit route. The unbinding mechanism is favored by two charged residues of the MA channel (Asp403 and Arg486, which are shown in cyan sticks) that facilitate the passage of the substrate through the MA channel, H-bonding to the polar head group of the substrate. The oleamide unbinding occurs in both FAAH monomers (at ~425 ns in mnr-A and at ~350 ns in mnr-B). The MA (red) and AB (orange) channels are depicted in molecular surface representation. The mutated “dynamic paddle” residues Ala432 (green) and Ala531 (green) are shown in space-filling representation. The enzymatic framework is shown in gray ribbons, while the lipids of the membrane are represented in cyan lines. Water molecules accessing the MA channel are also shown as sticks and balls. (TIF) [file pcbi.1004231.s010.tif]

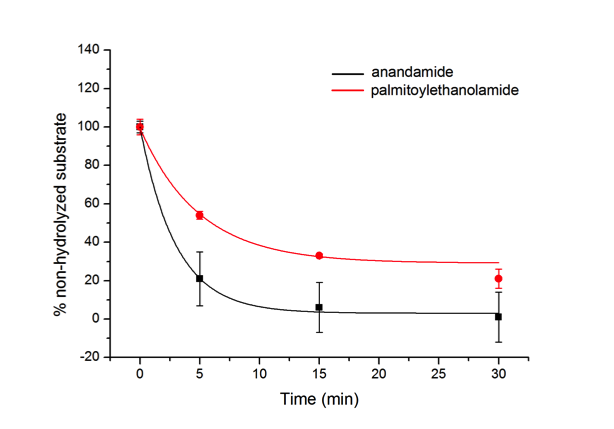

Supplement: S11 Fig — The reactions were quenched at different time points with the addition of four volumes of cold acetonitrile. Each set of data was fitted using simple exponential decay functions, whose parameters are reported in S7 Table in S2 Text. (TIF) [file pcbi.1004231.s011.tif]

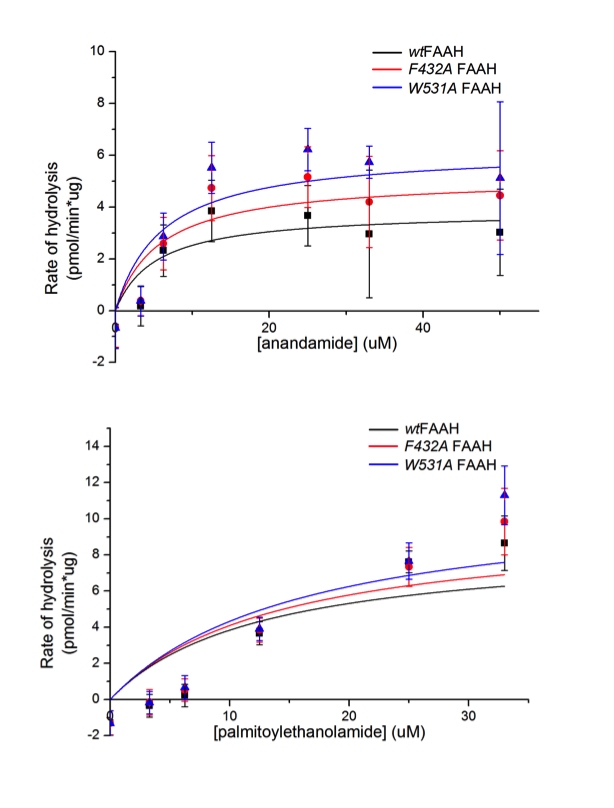

Supplement: S12 Fig — Reaction was stopped after 30 minutes at 37°C assuming that a steady state was reached (Michaelis Menten condition). Each set of data was fitted using a Michaelis Menten model. Details are reported in the main text. (TIF) [file pcbi.1004231.s012.tif]

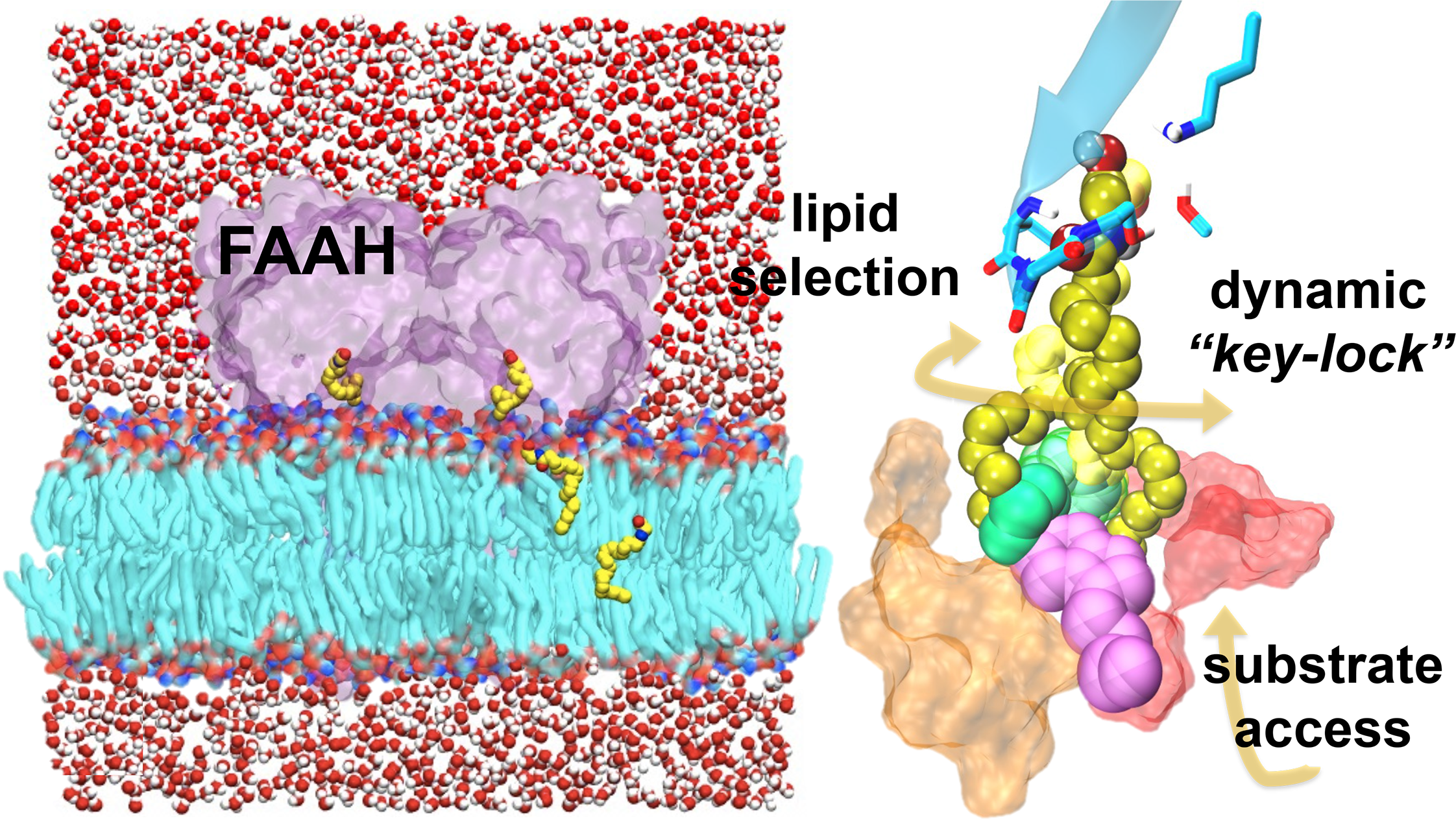

Supplement: S13 Fig — (TIF) [file pcbi.1004231.s013.tif]
