# Supplementary material for: Keys to Lipid Selection in Fatty Acid Amide Hydrolase Catalysis: Structural Flexibility, Gating Residues and Multiple Binding Pockets
Source: PLoS Comput Biol. 2015 Jun 25;11(6):e1004231. doi: 10.1371/journal.pcbi.1004231 (PMC4481349; doi:10.1371/journal.pcbi.1004231)
Supplement: S1 Text — Full docking details. Details on the conformational analysis of the lipid substrates. Definition of catalytically competent conformational states of the FAAH/substrate complexes. Reproducibility of the results in both FAAH subunits. Details on the location of the substrates within FAAH active site during MD. Details on the occurrence of pre-reactive conformational states during MD. Unbinding of oleamide in the mutFAAH system. Binding free energy calculations. (DOCX) [file pcbi.1004231.s014.docx]

**Supplementary Text 1: Supplementary text section**

**Keys to lipid selection in fatty acid amide hydrolase catalysis: Structural flexibility, gating residues and multiple binding pockets**

Giulia Palermo,^1^ Inga Bauer,^2^ Pablo Campomanes,^3^ Andrea Cavalli,^2,4^

Andrea Armirotti,^5^ Stefania Girotto,^2^ Ursula Rothlisberger^3^ and Marco De Vivo^1*^

1. Laboratory of Molecular Modeling and Drug Discovery, Istituto Italiano di Tecnologia, Genova, Italy
2. CompuNet, Istituto Italiano di Tecnologia, Genova, Italy

3. Laboratory of Computational Chemistry and Biochemistry,

Institute of Chemical Sciences and Engineering,

École Polytechnique Fédérale de Lausanne,

Lausanne, Switzerland

4. Department of Pharmacy and Biotechnology, University of Bologna, Bologna, Italy

5. D3-PharmaChemistry, Istituto Italiano di Tecnologia, Genova, Italy

*Corresponding authors:

marco.devivo@iit.it

1. **Docking studies**

All docking calculation were performed using the Autodock 4.2 software package.[35] In accordance to the catalytic mechanism of FAAH, the Lys142 was assumed as deprotonated. The charges of the deprotonated Lys142 was added as reported within the AMBER force field.[38,52] For all the docking calculations, a grid box size of 82 x 88 x 90 with spacing of 0.375 Å between the grid points was used. The grid box was centered between the Ser241 - Ser217 - Lys142 catalytic triad and the oxyanion hole (Ser241, Gly240, Gly239), covering the catalytic center surface of FAAH. In order to obtain a representative conformational space during the docking calculations, all the sigma bonds of the oleamide and palmitoylethanolamide (PEA), as well as the amide bonds, were treated as active torsional bonds. Twenty calculations consisting of 256 runs were performed, obtaining 5120 structures (256 x 20). The Lamarkian genetic algorithm was used. An initial population of 450 randomly placed individuals; a maximum number of 2,500,000 energy evaluations and a maximum number of 27,000 generations were considered. A mutation rate of 0.02 and a crossover rate of 0.8 were used. Results were clustered together differing about 2 Å in positional RMSD to evaluate them with respect to the free energy of binding. Docking data are reported in S1 Table. The initial binding mode of the studied substrates – i.e., anandamide, oleamide and PEA – is shown in S1 Fig.

1. **Set-up and equilibration of the protein/membrane complex**

A large membrane patch consisting of 571 POPE lipids was solvated in a TIP3P[37] water box of 150 Å x 95 Å x 130 Å (~182.600 total atoms) and equilibrated for ~30 ns, following the procedure described above. Then, the equilibrated membrane was used for the embedding of the FAAH protein in complex with its lipid substrates. FAAH has been embedded in the membrane by carefully following the procedure recommended by Tieleman in: Kandt C, Ash WL, Tieleman DP (2007) Methods 41: 475-488. In detail, after having oriented the protein within the lipid bilayer, the lipids overlapping with the protein within a cut-off radius from the protein atoms of 0.8–1.6 Å have been deleted. Importantly, both the FAAH transmembrane residues and the transversal α18/α19 helices were inserted into the membrane, as indicated by Bracey et al.[1] Moreover, although FAAH active site is not embedded in the membrane, Phe432 and Trp531 belong to the α19 helix, which is partially inserted into the membrane. Thus, the lipid bilayer is crucial for the dynamics of these residues. Then, the obtained protein/membrane complex has been equilibrated until reaching the convergence of important stability parameters of the protein (i.e., RMSD). Mechanical and physico-chemical properties of the POPE membrane were evaluated in terms of: *(i)* average area per lipid, *(ii)* lateral self-diffusion coefficient. Furthermore, during MD simulations, the stability of the residues inserted into the POPE membrane was ensured analyzing crucial helix properties (i.e., RMS deviation from ideal helix, helix radius, twist, and rise per residue). Full details on the set-up and equilibration of the protein/membrane complex are reported in our paper on the anandamide binding in FAAH.[25]

1. **Conformational analysis of the substrates**

Conformational changes of anandamide during dynamics were identified applying the Applegate and Glomset notation.[53-55] Accordingly, the anandamide conformations depend on three pairs of torsion angles along its arachidonoyl chain. Referring to the carbon numbering for anandamide shown in S2A Fig., the torsion angles involved are defined as: ω_5_ = C_5_-C_6_-C_7_-C_8_, ω_6_ = C_6_-C_7_-C_8_-C_9_, ω_8_ = C_8_-C_9_-C_10_-C_11_, ω_9_ = C_9_-C_10_-C_11_-C_12_, ω_11_ = C_11_-C_12_-C_13_-C_14_, ω_12_ = C_12_-C_13_-C_14_-C_15_. When two adjacent angles (e.g., the couple ω_5_–ω_6_) have the same sign ($\pm\pm$), either positive or negative, that region of the acyl chain tends to be elongated, whereas when the two angles have opposite sign ($\pm\mp$), a curvature is introduced in that region of the acyl chain. Dihedrals with angles from 0° to 180° are denoted with positive sign, while those from 180° to 360° are denoted with negative sign (as from 0° to -180°). The 0° reference angle corresponds to eclipsed substituents along the central bond axis. Following this notation, the anandamide molecule can assume six different shapes (S2A Fig.),[53,55] namely: (*i*) the extended (*Ex*) shape where the three pairs of angles are characterized by the same sign [($\pm\pm$)($\pm\pm$)($\pm\pm$)], resulting in a completely elongated anandamide acyl chain; (*ii*) the extended U (*Uex*) shape, where only the central ω_8_-ω_9_ has an opposite sign [($\pm\pm$)($\pm\mp$)($\pm\pm$)]; (*iii*) the J (*J*) shape, which shows a “hook” at the end of the acyl chain [($\pm\pm$)($\pm\pm$)($\pm\mp$)]; (*iv*) the parent J’ (*J’*) shape, where a “hook” is observed near the polar anandamide head-group [($\pm\mp$)($\pm\pm$)($\pm\pm$)]; (*v*) the U (*U*) shape, where the ω_5_-ω_6_ and ω_11_‒ω_12_ pairs have opposite sign [($\pm\mp$)($\pm\pm$)($\pm\mp$)]; (*vi*) and the helical (*Hx*) shape where each couple of angles has opposite sign [($\pm\mp$)($\pm\mp$)($\pm\mp$)]. In addition, we have identified two unique anandamide conformations within the FAAH protein.[25] These structures are characterized by two curvatures in the anandamide acyl chain and we named them: (*vii*) “half helical A” (*Hx/A*) [($\pm\pm$)($\pm\mp$)($\pm\mp$)] and (*viii*) “half helical B” (*Hx/B*) [($\pm\mp$)($\pm\mp$)($\pm\pm$)] shapes. The 8 anandamide conformations can be classified in three classes: the *“elongated” Ex* and *Uex* shapes, (class A), the *“hooked”* *J* and *J’* shapes (class B) and the *“curved” U*, *Hx*, *half-Hx/A* and *half-Hx/B* shapes (class C).

Conformational changes of oleamide during dynamics were classified via the same method. Referring to the carbon numbering for oleamide shown in S2B Fig., we have considered the ω_6_ = C_6_-C_7_-C_8_-C_9_, ω_7_ = C_7_-C_8_-C_9_-C_10_ and ω_9_ = C_9_-C_10_-C_11_-C_12_, ω_10_ = C_10_-C_11_-C_12_-C_13_ dihedrals. The ω_6_–ω_7_ and ω_9_–ω_10_ couples determine the curvature of the oleoyl chain in the regions adjacent to the Δ^9^ unsaturation. Four oleamide conformations were identified (S2B Fig.), namely: (*i’*) the extended (*Ex*) shape where the two pairs of angles have the same sign [($\pm\pm$)($\pm\pm$)]; (*ii’*) the J (*J*) shape that shows a “hook” at the end of the oleoyl chain [($\pm\pm$)($\pm\mp$)]; (*iii’*) the J’ (*J’*) shape, in which the “hook” is near the polar oleamide head-group [($\pm\mp$)($\pm\pm$)]; (*vi’*) and the helical (*Hx*) shape where both the two couples of ω dihedrals have opposite sign [($\pm\mp$)($\pm\mp$)]. The four oleamide shapes were classified in three classes: class of the elongated shapes (class A), which is composed by the *Ex* oleamide shape, class of the *“hooked”* (hairpin like) *J* and *J’* shapes (class B) and the class of the curved shapes (class C) that is composed by the *Hx* oleamide shape.

Due to the lack of unsaturations within the PEA acyl chain, the PEA conformational changes were analyzed considering the changes in length of the lipid with respect to the initial configuration, which is characterized by an extended shape (*Ex*). In detail, initially, the distance (*l*_PEA_) between the PEA carbonyl carbon and the last PEA atom is 16.5 Å. If *l*_PEA_ *>* 14 Å, the substrate is extended (*Ex* – class A). If *l*_PEA_ *<* 12 Å, PEA is fully curved (*Hx* – class C), whereas for intermediate values (i.e., 12 Å < *l*_PEA_ < 14 Å), PEA assumes *“hooked”* (*J* – class B) conformations (S2C Fig.). In all conformational analysis, the RMSD from the initial configuration of anandamide, oleamide and PEA was used for comparison (S5 and S6 Figs.).

1. **Catalytically competent conformational states of the FAAH/substrate complexes**

The pre-organization of the FAAH active site for the substrates hydrolysis has been identified via the definition of catalytically competent conformational states of the FAAH/substrate complexes that are characterized by optimal distances and orientations of key structural parameters involved in the enzymatic reaction. In detail, the key geometrical descriptors (S7 Fig.) that have been used to define the catalytically competent conformational states of the FAAH/substrate complex are: (1) the distance between the substrate carbonyl carbon and the oxygen of Ser241 (C@substrate-O@Ser241), *d_1_*, lower than 3.4 Å; (2) the donor-acceptor H-bond distances formed by the FAAH catalytic triad, *d_2_* and *d_3_*, lower than 3 Å; (3) the attacking angle formed by the nucleophilic species (O@Ser241) and the substrate carbonyl plane *θ_1_* of about 110° ± 20° (i.e., *θ_1_* corresponds to the so-called *Bürgi-Dunitz trajectory*, as described in Burgi HB, Dunitz JD, Shefter E (1973). J Am Chem Soc 95: 5065-5067); (4) the H-bond angles defined as the proper angles between the donor, the hydrogen and acceptor atoms, *θ_2_* and *θ_3_*, equal to 180° ± 20°. The *θ_1_*, *θ_2_*, *θ_3_*, angles were described from 0° to 360°. For simplicity, catalytically competent conformational states of the FAAH/substrate complexes are here referred as pre-reactive conformations, as well.

1. **Reproducibility of the results in both FAAH subunits**

As reported in the body of the paper, the two FAAH subunits show a similar behavior in terms of location of the substrate and occurrence of pre-reactive conformations. Due to the reproducibility of the results in both monomers, statistics was accumulated for the aggregate monomers on a total of sampling time of ~700 ns (i.e., ~70,000 frames) for each system (~350 ns per monomer – see the Methods section for details). S2, S3 and S4 Tables report the statistical analysis for each monomer of all the six studies systems, namely: (1) *wt*-FAAH/anandamide; (2) *wt*-FAAH/oleamide; (3) *wt*-FAAH/palmitoylethanolamide; (4) *mut*-FAAH/anandamide; (5) *mut*-FAAH/oleamide and (6) *mut*-FAAH/palmitoylethanolamide.

1. **Details on the location of the substrates within FAAH active site during MD**

***wt*FAAH/anandamide** **system.** During classical MD, when initially located in the MA channel, anandamide reversibly transfers its arachidonoyl chain to the adjacent AB channel.[1,25] In monomer-A (mnr-A), the lipid is very stable in the MA channel for the first ~200 ns of MD, including the equilibration time (Fig. 2 and S8 Fig.). Afterwards, it mostly locates at the MA/AB interface region – i.e., transition (T) region – and remains in the AB channel between ~325/330 ns, ~350/355 ns, ~400/405 ns and ~465/480 ns. In monomer B (mnr-B), anandamide moves to the T region during the equilibration time and stays at the channel boundary for most of the simulation, showing one MA–>AB transition between ~380/390 ns.

***wt*FAAH/oleamide** **system.** In this system, oleamide reversibly transfers its acyl chain from the MA to the AB channel, as well. In both the enzymatic subunits, the lipid shows a major tendency to occupy the MA channel (Fig. 2 and S8 Fig.). In mnr-A, oleamide reaches the T region during the equilibration time and transfers its acyl chain in AB only for short times (~330/338 ns and ~475/490 ns). The lipid switches back from the AB to MA channel between ~340/435 ns. In mnr-B, oleamide preferentially locates into the MA channel.

***wt*FAAH/palmitoylethanolamide system.** Along the simulations, MA<->AB transfers of the palmitoylethanolamide acyl chain do not occur. The lipid is mainly located in the MA channel of both the enzyme subunits (Fig. 2 and S8 Fig.).

***mut*FAAH/anandamide** **system.** We detect several MA<–>AB transitions of the arachidonoyl chain. In mnr-A, anandamide locates in the MA channel approximately between ~0/215 ns and ~245/260 ns, remains at the MA/AB interface region between ~240/245 ns and lives within the AB channel between ~215/240 ns and ~260/500 ns (S9 Fig.). In mnr-B, the lipid mainly locates in the MA channel with few transitions in the T region and within the AB channel.

***mut*FAAH/oleamide system.** During MD, oleamide never transfers its acyl chain from the MA channel into the adjacent AB channel (S9 Fig.). Indeed, the lipid remains for most of the production run within the MA channel in both FAAH subunits, locating in the MA/AB interface region just for short times (~340/345 ns in mnr-A; ~150/160 ns and ~225/240 ns in mnr-B).

***mut*FAAH/palmitoylethanolamide system.** Palmitoylethanolamide transfers its palmitoyl chain from the MA to the AB channel in the early phase of the equilibration (~10/20 ns), in both FAAH monomers (S9 Fig.).

1. **Details on the occurrence of pre-reactive conformational states during MD**

Pre-reactive (i.e., catalytically competent conformations) and non-reactive states of the FAAH/substrate complexes, sampled over the production runs are reported in S2 Table and Fig. 3B of the main text. Percentages of pre-reactive conformations in the MA, T and AB regions of FAAH active site are also reported in S3 Table. Data are reported for each FAAH monomer and for the aggregate monomers as well. As widely discussed in the main text, pre-reactive conformations are sampled for the 26.5 %, 15.7 % and the 10.5 % in the *wt*FAAH/anandamide, *wt*FAAH/oleamide and *wt*FAAH/PEA systems, respectively. Pre-reactive conformations are not sampled in the *mut*FAAH/anandamide and in the *mut*FAAH/oleamide systems, thus highlighting the crucial role of Phe432 and Trp531 in inducing specific conformations of the unsaturated lipids that are prone to be hydrolyzed. In the *mut*FAAH/PEA system, pre-reactive conformations are the 14.7 % of the production run.

It is important to note that, the low percentage of pre-reactive states with respect to the non-reactive states is in agreement with the previous results published by Lodola et al.[31] The authors have shown that pre-reactive conformations are rarely sampled during MD, suggesting that FAAH reactivity could be dominated by distinct high energy and lowly populated conformations.

1. **Unbinding of oleamide in the *mut*FAAH system**

As reported in the main text, in absence of the *“dynamic paddle”* residues, oleamide spontaneously unbinds from FAAH active site and locates within the lipid bilayer at ~425 ns in mnr-A and at ~350 ns in mnr-B (S10 Fig.). Oleamide departs from FAAH active site using the MA channel as an exit route. The unbinding occurs via the same mechanism in the two FAAH subunits and is favored by two charged residues (Asp403–Arg486) that facilitate the passage of the substrate through the MA channel, H-bonding to the polar head group of the substrate. The mutation of Phe432, which in the *wt*-system interacts with the oleamide Δ^9^ double bond, causes a destabilization of the oleoyl chain within active site. In addition, oleamide is a primary amide that, therefore, does not have the ability to form H-bond interactions with the CP residue Thr236, which has been shown to be critical for leaving group departure, after substrate hydrolysis. ^1,13,15^

1. **Binding free energy calculations**

Binding free energies (Δ*G*_Bind_) for the three ligands in the wild type (*wt*) and mutated (*mut*) FAAH systems were estimated by combining the Thermodynamic Cycle and MD simulations. This is a post-processing method in which representative snapshots from the ensemble of conformations are used to calculate the free energy change between two states (i.e., the bound and free state of the FAAH protein and the substrates). The free energy is calculated by decomposing the contributions coming from the solvation energy from the reactants and products and from the binding free energy *in vacuo*. The MM/PBSA (Molecular Mechanics/Poisson-Boltzmann Surface Area)[50,51] method implemented in the Amber 12 package[52] was used to calculate the solvation energies by solving the Linearized Poisson–Boltzmann equation. This method has been shown to be an efficient computational approach for the estimation of the binding affinities in several protein/ligand complexes (such as in: Collu F, Vargiu AV, Dreier J, Cascella M, Ruggerone P (2012) J. Am. Chem. Soc.134: 19146-19158). Accordingly, the Δ*G*_Bind_ between a protein and a ligand to form a protein/ligand complex can be calculated as in eq [2].

Δ*G*_Bind_ = Δ*E*_MM_ + Δ*G*_Sol_ – *T*Δ*S* [2]

Δ*E*_MM_ = Δ*E*_internal_ + Δ*E*_electrostatic_ + Δ*E*_vdW_ [3]

Δ*G*_Sol_ = Δ*G*_SA_ + Δ*G*_PB_  [4]

where ΔE_MM_ is the total gas phase energy, given by the sum of Δ*E*_internal_, Δ*E*_electrostatic_, Δ*E*_vdW_. Δ*E*_internal_ is the internal energy arising from bond, angle and dihedral terms in the MM force field. Δ*E*_electrostatic_ and Δ*E*_vdW_ are the electrostatic and van der Waals contributions as calculated by the MM force field. Δ*G*_Sol_ is the sum of the nonpolar (Δ*G*_NP_) and polar (Δ*G*_PB_) contributions to the solvation free energy. Δ*G*_PB_ is computed in continuum solvent, using the Poisson–Boltzmann model. Δ*G*_SA_ can be derived from the solvent-accessible surface area (SA). The last term in eq [2] (*T*Δ*S*) is the conformational entropy upon binding computed by normal-mode analysis.

For the MM/PBSA calculations, 5000 equally spaced snapshots of each of the six simulated systems were extracted from the equilibrated MD trajectories (i.e., the last ~350 ns; ~35,000 frames). In order to get uncorrelated structures, we extracted one snapshot every 70 ps from all the six ~350 ns trajectories. All water molecules and counterions were removed before MM/PBSA calculations. The dielectric constants ε = 1 and ε = 80 were used to reproduce the *in vacuo* and in solvent conditions, respectfully. It is important to mention that, while all MD simulations have been carried out using GROMACS 4,[41] MM/PBSA calculations have been performed using AMBER 12.[52] MM/PBSA is a post-processing method implemented in AMBER 12 that allows calculating the free energy change between states starting from representative snapshots of the ensemble of conformations. Given the consistence of the here employed force-field parameters, the configurations obtained with GROMACS 4 are suitable for the post-processing analysis carried out with AMBER 12. Indeed, MD trajectories in the GROMACS 4 format have been converted in AMBER format, while maintaining the force-field parameters.

The calculated Δ*G*_Bind_ for the anandamide, oleamide and PEA substrates, which must be considered only as a qualitative indication of binding affinity, in the *wt* and *mut* FAAH protein systems are reported in S4 Table. The different components of Δ*G*_Bind_ are also reported in S5 Table.

As a result, in the *wt*-FAAH/anandamide system, anandamide shows a remarkable binding affinity for the *wt* protein (Δ*G*_Bind_ = -43.00 ± 0.74 kcal/mol) and a lower affinity for the *mut* FAAH (Δ*G*_Bind_ = -29.61 ± 4.26 kcal/mol). Indeed, the difference of Δ*G*_Bind_ between the *wt* and the *mut* systems (ΔΔ*G*_Bind_) is -13.39 kcal/mol, highlighting the crucial role of Phe432 and Trp531 in the substrate binding. In the case of the *wt*FAAH/oleamide system, the Δ*G*_Bind_ is -37.88 ± 0.09 kcal/mol. The data supports the lower specificity of FAAH for oleamide, with respect anandamide.[9,10] The Δ*G*_Bind_ of the *mut*FAAH/olemide system is -32.38 ± 0.10 kcal/mol with a ΔΔ*G*_Bind_ between the *wt* and the *mut* systems of -5.49 kcal/mol. As in the case of anandamide, the *“dynamic paddle”* seems to be responsible of the higher affinity of oleamide for the *wt* protein, with respect the *mut* FAAH. In the case of the *wt*FAAH/PEA system, the substrate shows a low binding affinity, compared to the *wt*FAAH/anandamide and the *wt*FAAH/oleamide systems, as the calculated Δ*G*_Bind_ is -32.37 ± 0.55 kcal/mol. The data is in agreement with the significant lower selectivity of FAAH for PEA with respect to the unsaturated lipids.[9,10] In the *mut*FAAH/PEA system, the substrate shows a higher affinity for the *mut* FAAH, with respect the *wt* protein, as the calculated Δ*G*_Bind_ for the *mut*FAAH/PEA system is -39.34 ± 1.66 kcal/mol, with a ΔΔ*G*_Bind_ between the *wt* and the *mut* systems of 6.97 kcal/mol. As reported in the body of the paper and in these SI, the total percentage of catalytically significant conformations of PEA is the 10.5 % and 14.7 % in the *wt* FAAH and *mut* FAAH systems, respectively. These data suggest that FAAH seems to be more specific for PEA in absence of the *“dynamic paddle”* that, contrariwise, seems to be crucial for the binding of the unsaturated lipids (anandamide and oleamide) within FAAH. Binding free energies (*Δ*G_Bind_ – see Methods section and SI), obtained via the MM/PBSA method,[50,51] confirm the highest affinity of anandamide for the *wt*FAAH. Oleamide’s affinity for FAAH is ~5 kcal mol^-1^ lower than that found for anandamide (see the main text). PEA shows ~11 kcal mol^-1^ lower affinity for FAAH, compared to anandamide. This energetic difference well agrees with the non-equilibrium conditions of our competition experiments, confirming a high preference of FAAH for its primary substrate (i.e. anandamide).
