# Supplementary material for: Keys to Lipid Selection in Fatty Acid Amide Hydrolase Catalysis: Structural Flexibility, Gating Residues and Multiple Binding Pockets
Source: PLoS Comput Biol. 2015 Jun 25;11(6):e1004231. doi: 10.1371/journal.pcbi.1004231 (PMC4481349; doi:10.1371/journal.pcbi.1004231)
Supplement: S2 Text — (DOCX) Supporting tables including docking data (S1 Table), statistical analysis of the occurrence of catalytically relevant conformations (S2-S3 Tables), binding free energies (ΔG Bind—S4 Table) and ΔG Bind energetic contributions (S5 Table). Supporting data about competition assays are also reported (S6-S7 Tables). (DOCX) [file pcbi.1004231.s015.docx]

**Supplementary Text 2:**

**Supplementary information including S1-S7 Tables.**

**Keys to lipid selection in fatty acid amide hydrolase catalysis: Structural flexibility, gating residues and multiple binding pockets**

Giulia Palermo,^1^ Inga Bauer,^2^ Pablo Campomanes,^3^ Andrea Cavalli,^2,4^

Andrea Armirotti,^5^ Stefania Girotto,^2^ Ursula Rothlisberger^3^ and Marco De Vivo^1*^

1. Laboratory of Molecular Modeling and Drug Discovery,

Istituto Italiano di Tecnologia, Via Morego 30, 16163 Genova, Italy

1. CompuNet, Istituto Italiano di Tecnologia, Via Morego 30, 16163 Genova, Italy

3. Laboratory of Computational Chemistry and Biochemistry,

Institute of Chemical Sciences and Engineering,

École Polytechnique Fédérale de Lausanne,

CH-1015 Lausanne, Switzerland

4. Department of Pharmacy and Biotechnology, University of Bologna,

Via Belmeloro 6, I-40126 Bologna, Italy

5. D3-PharmaChemistry, Istituto Italiano di Tecnologia, Via Morego 30, 16163 Genova, Italy

*Corresponding authors:

marco.devivo@iit.it

**Tables**

|  | FAAH/OA | FAAH/PEA |
| --- | --- | --- |
| Estimated free energy of binding [(1)+(2)+(3)-(4)] | -6.56 | -6.78 |
| Dissociation constant Kd [µM] | 15.51 | 10.68 |
| Final intermolecular energy (1) | -11.04 | -11.85 |
| vdW + Hbond + Dessolv energy | -10.85 | -11.78 |
| Electrostatic energy | -0.18 | -0.07 |
| Total internal (2) | -0.75 | -0.99 |
| Torsional free energy (3) | 4.47 | 5.07 |
| Unbound system energy (4) | -0.75 | -0.99 |

**S1 Table.** Full docking data (energies and dissociation constant (Kd) values, reported in rows) for the final docked FAAH/oleamide (second column) and FAAH/PEA (third column) complexes. The dissociation constant (Kd) is expressed as µM (micromolar) and is measured at 298.15 K. Energies are expressed in kcal/mol.

**S2 Table.** Percentages of pre-reactive/non-reactive states (in rows) of the *wt*FAAH/anandamide (a), *wt*FAAH/oleamide (b), *wt*FAAH/PEA (c), *mut*FAAH/anandamide (d), *mut*FAAH/oleamide (e) and *mut*FAAH/PEA (f) systems, shown for monomer-A (Mnr-A, second column) and monomer-B (Mnr-B, third column). The aggregate results over both FAAH monomers (mnr-A/B) are also reported in the fourth column. Percentages were calculated for the equilibrated systems (after ~150 ns).

| **(a) *wt*FAAH/anandamide** | | | |
| --- | --- | --- | --- |
|  | **Mnr-A** | **Mnr-B** | **Mnr-A/B** |
| **catalytic** | 12.1 % | 40.9 % | 26.5 % |
| **non-catalytic** | 87.9 % | 59.1 % | 73.5 % |
|  |  |  |  |
| **(b) *wt*FAAH/oleamide** | | | |
|  | **Mnr-A** | **Mnr-B** | **Mnr-A/B** |
| **catalytic** | 19.1 % | 12.2 % | 15.7 % |
| **non-catalytic** | 80.9 % | 87.8 % | 84.3 % |
|  |  |  |  |
| **(c) *wt*FAAH/PEA** | | | |
|  | **Mnr-A** | **Mnr-B** | **Mnr-A/B** |
| **catalytic** | 11.7 % | 9.4 % | 10.5 % |
| **non-catalytic** | 88.3 % | 90.6 % | 89.5 % |
|  |  |  |  |
| **(d) *mut*FAAH/anandamide** | | | |
|  | **Mnr-A** | **Mnr-B** | **Mnr-A/B** |
| **catalytic** | 0.0 % | 0.0 % | 0.0 |
| **non-catalytic** | 100.0 % | 100.0 % | 100.0 |
|  |  |  |  |
| **(e) *mut*FAAH/oleamide** | | | |
|  | **Mnr-A** | **Mnr-B** | **Mnr-A/B** |
| **catalytic** | 0.0 % | 0.2 % | 0.1 % |
| **non-catalytic** | 100.0 % | 99.8 % | 99.9 % |
|  |  |  |  |
| **(e) *mut*FAAH/PEA** | | | |
|  | **Mnr-A** | **Mnr-B** | **Mnr-A/B** |
| **catalytic** | 11.7 % | 9.4 % | 10.5 % |
| **non-catalytic** | 88.3 % | 90.6 % | 89.5 % |

**S3 Table.** Percentages of pre-reactive conformations in the MA (second column), T (third column) and AB (fourth column) regions, shown for monomer A (Mnr-A, second row) and monomer B (Mnr-B, third row) of the *wt*FAAH/anandamide (a), *wt*FAAH/oleamide (b), *wt*FAAH/PEA (c) and *mut*FAAH/PEA (d) systems systems. The aggregate results over both monomers (mnr-A/B) are also shown in the last row. Data for the *mut*FAAH/anandamide and *mut*FAAH/oleamide systems are not reported, since catalytically relevant conformations were not detected in these systems.

| **(a)  *wt*FAAH/anandamide** | | | |
| --- | --- | --- | --- |
|  | **MA** | **T** | **AB** |
| **Mnr-A** | 19.2 % | 73.0 % | 7.8 % |
| **Mnr-B** | 23.0 % | 70.2 % | 6.8 % |
| **Mnr-A/B** | 21.1 % | 71.6 % | 7.3 % |
| **(b)  *wt*FAAH/oleamide** | | | |
|  | **MA** | **T** | **AB** |
| **Mnr-A** | 77.3 % | 22.9 % | 0.03 % |
| **Mnr-B** | 78.7 % | 18.8 % | 2.4 % |
| **Mnr-A/B** | 78.0 % | 20.9 % | 1.2 % |
|  |  |  |  |
| **(c)  *wt*FAAH/PEA** | | | |
|  | **MA** | **T** | **AB** |
| **Mnr-A** | 81.1 % | 19.0 % | 0.0 % |
| **Mnr-B** | 92.7 % | 7.3 % | 0.0 % |
| **Mnr-A/B** | 86.9 % | 13.1 % | 0.0 % |
| **(d)  *mut*FAAH/PEA** | | | |
|  | **MA** | **T** | **AB** |
| **Mnr-A** | 0.0 % | 23.1 % | 76.9 % |
| **Mnr-B** | 0.0 % | 3.9 % | 96.0 % |
| **Mnr-A/B** | 0.0 % | 13.5 % | 86.5 % |

**S4 Table.** Binding free energies (Δ*G*_Bind_) for the anandamide, oleamide and PEA substrates (in rows) in the *wt* (second column) and *mut* (third column) FAAH proteins. The difference of Δ*G*_Bind_ between the *wt* and *mut* systems (ΔΔ*G*_Bind_) is reported in the fourth column. Energies are expressed in kcal/mol.

|  | **Δ*G*_Bind_**  ***wt*FAAH** | **Δ*G*_Bind_**  ***mut*FAAH** | **ΔΔ*G*_Bind_**  ***wt*FAAH*–mut*FAAH** |
| --- | --- | --- | --- |
| **anandamide** | -43.00 ± 0.74 | -29.61 ± 4.26 | -13.39 |
| **oleamide** | -37.88 ± 0.09 | -32.38 ± 0.10 | -5.49 |
| **palmitoylethanolamide** | -32.37 ± 0.55 | -39.34 ± 1.66 | 6.97 |

**S5 Table.** Components of Δ*G*_Bind_, reported for the *wt*FAAH (second row) and the *mut*FAAH complexes (third row). Δ*E*_vdW_ is the van der Waals contribution from Molecular Mechanics (MM). Δ*E*_electrostatic_ is the electrostatic energy as calculated from the MM force field. Δ*G*_PB_ and Δ*G*_NP_ are the polar and non polar contributions to the solvation free energy. Δ*E*_internal_ is the energy arising from bond, angle, dihedral terms in the MM force field (this term always amounts to zero in a single trajectory approach). Δ*G*_Bind_ is also reported. More details are reported in the main text. Energies are expressed in kcal/mol.

| Energies [kcal/mol] | ***wt*FAAH/**  **anandamide** | ***mut*FAAH/**  **anandamide** |
| --- | --- | --- |
| Δ*E*_vdW_ | -55.17 ± 0.73 | -46.76 ± 4.26 |
| Δ*E*_electrostatic_ | -40.32 ± 0.08 | -26.46 ± 0.09 |
| Δ*G*_PB_ | 57.37 ± 0.07 | 48.68 ± 0.07 |
| Δ*G*_NP_ | -4.88 ± 0.00 | -5.07 ± 0.00 |
| **Δ*G*_Bind_** | **-43.00 ± 0.74** | **-29.61 ± 4.26** |
|  |  |  |
|  | ***wt*FAAH/**  **oleamide** | ***mut*FAAH/**  **oleamide** |
| Δ*E*_vdW_ | -45.51 ± 0.08 | -43.00 ± 0.12 |
| Δ*E*_electrostatic_ | -14.95 ± 0.06 | -15.50 ± 0.11 |
| Δ*G*_PB_ | 26.51 ± 0.06 | 30.37 ± 0.12 |
| Δ*G*_NP_ | -3.95 ± 0.00 | -4.30 ± 0.01 |
| **Δ*G*_Bind_** | **-37.88 ± 0.09** | **-32.38 ± 0.10** |
|  |  |  |
|  | ***wt*FAAH/**  **PEA** | ***mut*FAAH/**  **PEA** |
| Δ*E*_vdW_ | -49.01 ± 0.55 | -51.68 ± 1.6 |
| Δ*E*_electrostatic_ | -23.07 ± 0.07 | -24.33 ± 0.20 |
| Δ*G*_PB_ | 44.41 ± 0.08 | 41.26 ± 0.20 |
| Δ*G*_NP_ | -4.70 ± 0.00 | -4.60 ± 0.06 |
| **Δ*G*_Bind_** | **-32.37 ± 0.55** | **-39.34 ± 1.66** |

**S6** **Table**. Exponential decay parameters obtained from the fitting of the competition assay data sets reported in Fig. 6.

y = A_1_ e(-x/t_1_) + y_0_ (Origin Pro 8.6)

| anandamide | y_0_ | A_1_ | t_1_ | 1/t_1_ |
| --- | --- | --- | --- | --- |
| WT | 9.54 | 90.43 | 2.73 | 0.366 |
| F432A | 23.01 | 77.94 | 5.51 | 0.181 |
| W531A | 31.24 | 68.69 | 2.71 | 0.369 |

| PEA | y_0_ | A_1_ | t_1_ | 1/t_1_ |
| --- | --- | --- | --- | --- |
| WT | 13.59 | 86.46 | 15.33 | 0.065 |
| F432A | 10.71 | 89.22 | 7.20 | 0.139 |
| W531A | 29.57 | 70.36 | 3.18 | 0.314 |

**S7 Table**: Exponential decay parameters obtained from the fitting of the competition assays data sets reported in S11 Fig.

y = A_1_ e(-x/t_1_) + y_0_ (Origin Pro 8.6)

| Microsomes | y_0_ | A_1_ | t_1_ | 1/t_1_ |
| --- | --- | --- | --- | --- |
| anandamide | 3.07 | 96.93 | 2.99 | 0.334 |
| PEA | 29.16 | 70.19 | 4.94 | 0.202 |
